# New Variants Including *ARG1* Polymorphisms Associated with C-Reactive Protein Levels Identified by Genome-Wide Association and Pathway Analysis

**DOI:** 10.1371/journal.pone.0095866

**Published:** 2014-04-24

**Authors:** Nadimuthu Vinayagamoorthy, Hae-Jin Hu, Seon-Hee Yim, Seung-Hyun Jung, Jaeseong Jo, Sun Ha Jee, Yeun-Jun Chung

**Affiliations:** 1 Integrated Research Center for Genome Polymorphism, The Catholic University of Korea, College of Medicine, Seoul, Korea; 2 Department of Microbiology, The Catholic University of Korea, College of Medicine, Seoul, Korea; 3 Department of Medical Education, The Catholic University of Korea, College of Medicine, Seoul, Korea; 4 Institute of Health Promotion, Graduate School of Public Health, Yonsei University, Seoul, Korea; New Jersey Institute of Technology, United States of America

## Abstract

C-reactive protein (CRP) is a general marker of systemic inflammation and cardiovascular disease (CVD). The genetic contribution to differences in CRP levels remains to be explained, especially in non-European populations. Thus, the aim of this study was to identify genetic loci associated with CRP levels in Korean population. We performed genome-wide association studies (GWAS) using SNPs from 8,529 Korean individuals (7,626 for stage 1 and 903 for stage 2). We also performed pathway analysis. We identified a new genetic locus associated with CRP levels upstream of *ARG1* gene (top significant SNP: rs9375813, *P_meta_* = 2.85×10^−8^), which encodes a key enzyme of the urea cycle counteract the effects of nitric oxide, in addition to known *CRP* (rs7553007, *P_meta_* = 1.72×10^−16^) and *HNF1A* loci (rs2259816, *P_meta_* = 2.90×10^−10^). When we evaluated the associations between the CRP-related SNPs with cardiovascular disease phenotypes, rs9375813 (*ARG1*) showed a marginal association with hypertension (*P* = 0.0440). To identify more variants and pathways, we performed pathway analysis and identified six candidate pathways comprised of genes related to inflammatory processes and CVDs (*CRP, HNF1A*, *PCSK6, CD36*, and *ABCA1*). In addition to the previously reported loci (*CRP, HNF1A*, and *IL6*) in diverse ethnic groups, we identified novel variants in the *ARG1* locus associated with CRP levels in Korean population and a number of interesting genes related to inflammatory processes and CVD through pathway analysis.

## Introduction

C-reactive protein (CRP) is an acute phase reactant protein and a general marker of systemic inflammation that is produced by the liver. High CRP levels are known to be associated with cardiovascular disease (CVD) risk factors, including hypertension, coronary heart disease (CHD), and stroke, in addition to traditional risk factors such as BMI, smoking, diabetes, and cholesterol levels [1].

The heritability of CRP levels is estimated to be 25% to 40%, indicating that genetic variations can affect inter-individual or inter-ethnic group differences in CRP levels [2]. Indeed, CRP levels vary significantly among different ethnic groups [3]. For example, serum CRP levels were reported to be relatively lower in East Asians compared to Europeans, South Asians, and Aboriginal peoples in Canada [3]. Several large-scale genome-wide association studies (GWAS) to identify genetic links to difference in CRP levels have been undertaken; however, most of these studies were performed in European populations [4], [5]. In addition to the well-known variants that correlate with CRP levels in Europeans such as *CRP, HNF1A* (hepatic nuclear factor 1-alpha), and *APOE* (apolipoprotein E), some recent GWASs have identified new variants such as *IL6* (interleukin-6) in the Japanese population and *TREM2* (triggering receptors expressed by myeloid cells 2) in African American women [6], [7]. Differences in allele frequencies, linkage disequilibrium (LD), effect size, and biological adaptations may influence the identification of variants in different ethnic groups [8].

In spite of the identification of these CRP-associated single-nucleotide polymorphisms (SNPs) and genetic loci by large-scale GWASs, the genetic contributions to differences in CRP levels still need further investigation. Biological pathway-based analyses may be able to obtain more meaningful information from high-throughput whole genome data [8]. Pathway analysis can even suggest candidate variants that might be missed in a classical GWAS approach [9]. In the present study, we used a combined approach of GWAS and pathway analysis and attempted to identify SNPs associated with CRP levels in the Korean population.

## Materials and Methods

### Study subjects for stage 1 and stage 2 GWAS

As for the stage 1 subjects, a total of 7,626 individuals were used who have been participated in the Korea Association Resource project (KARE, stage I) [10]. Half of the subjects were recruited from one urban community (Ansan) and the other half came from one rural community (Ansung), Gyeonggi province, Korea. As for the stage 2 subjects, 903 independent samples were obtained from Yonsei University in Korea, which were genotyped with the same Affymetrix Genome-Wide Human SNP Array 5.0 platform. The general characteristics of stage 1 and stage 2 subjects including the CRP levels are summarized in Table S1 in File S1.

### Genotyping and quality control of the study population for stage 1 GWAS

The discovery subjects were genotyped using the Affymetrix Genome-Wide Human SNP Array 5.0. Genotypes were called using the BRLMM algorithm (http://media.affymetrix.com/support/technical/whitepapers/brlmm_whitepaper.pdf). Quality control was performed according to the previous studies [10], [11]. We adjusted the individual data with discordant sex information and filtered out those with the higher than 3% genotype failure rate. We also excluded individuals with the heterozygosity rate more than 3 standard deviations away from the mean. We applied SNP imputation to increase the coverage of variants by capturing additional association signals. Imputed SNP data were obtained from Cho *et al.* [10] who generated them using IMPUTE software [12] based on JPT/CHB data of HapMap as a reference panel. Based on these imputed SNP genotypes, we used standard quality control parameters such as SNP call rate >95%, minor allele frequency (MAF) >5% and Hardy-Weinberg equilibrium *P*>0.001. We excluded individuals with a CRP level greater than 10 mg/dL for our linear regression analysis. We also excluded participants with missing CRP levels and with diabetes according to the previous study [4]. Population stratification analysis of the phase 1 (KARE) data was already performed by Cho *et al.* [10] using principal component analysis and multidimensional scaling, in which no population stratification was observed. Through this quality control process, genotypes of 7,626 individuals for 1,219,546 autosomal SNPs were used for association analysis of the stage 1 cohorts.

### Stage 2 GWAS with the independent samples

To confirm the association of the identified SNPs from the stage 1 data, we analyzed an additional data set of 979 Korean individuals from Seoul area of South Korea, part of the Korean Metabolic Syndrome Research Initiative study [13]. We applied the same filtration process described above and selected 903 individuals whose CRP level and covariate information are available for subsequent analyses.

### Ethics statement

The local Ethics Committee approved this study, and written informed consent was obtained from all patients.This study was approved by the Institutional Review Board of the Catholic University of Korea School of Medicine (CUMC07U047).

### Statistical analysis

Linear regression analysis was performed assuming an additive model to determine the association of variants with CRP levels. Information on CRP levels and covariates were ascertained from KARE. CRP concentrations were transformed using the natural logarithm function to ensure a normal distribution. Linear regression analysis was performed assuming an additive model. Among the clinical covariates such as age, sex, body mass index, smoking, drinking, high-density lipoprotein (HDL) cholesterol, triglycerides, waist circumference, fasting glucose, average pulse, systolic blood pressure, diastolic blood pressure, and history of type 2 diabetes applied in the previous studies of CRP [14], we applied age, sex, body mass index, and average pulse for the regression analysis which are available in most individuals. We combined the stage 1 and 2 results by inverse-variance meta-analysis under the assumption of fixed effects. Statistical analyses were performed using PLINK [15]. We used Haploview (version 1.4) to create Manhattan plots and to calculate LD using a default distance option of 500 kb [16]. SNAP software was used to annotate the proxy of the top SNP [17]. In SNAP, a regional association plot was drawn with the following options: 1000 Genomes Pilot 1 SNP data set, CHB and JPT population panel, *r* ^2^ threshold of 0.8, and a distance limit between the query SNP and the proxy SNP of 500 kb. The statistical power of the study was evaluated using QUANTO version 1.2.4 [18]. In QUANTO, a gene only hypothesis was applied with continuous outcome from independent individuals using a desired type I error rate of 0.05 in 2-tailed test. The study had 90% power to detect association of a variant with a MAF = 0.02 and an effect size of 0.1 for additive model.

### SNP prioritization

SNP prioritization was performed via GWASrap (http://jjwanglab.org/gwasrap) [19]. This tool generates a re-prioritized genetic variant list by combining the original statistical value and variant prioritization score. The 13,345 GWAS SNPs with *P*<0.01 were applied as input values.

### Pathway and network analysis

We used ICSNPathway software to do pathway analysis and to identify candidate SNPs from our GWAS [20]. To get more reliable pathway analysis, we also used GSA-SNP software [21]. In ICSNPathway software, we chose the following options as follows; 500 kb up and downstream of the gene as a rule of mapping SNPs to the genes; a threshold of *P*<10^−3^ for extracting SNPs from GWAS; HapMap Han Chinese in Beijing (CHB) data was used for the imputation of HapMap population; 200 kb for distance for calculating LD. The rest of options were set as default in ICSNPathway software. As the database of pathways, we chose, Gene Ontology (GO) database. Pathways with false discovery rate (FDR) <0.05 and nominal *P*<0.05 were considered to be associated with CRP levels. In GSA-SNP software, based on the unimputed SNP *P*-values from GWAS, we applied GSA-SNP with the default parameters: k-th best SNP as 2, SNP-Gene mapping with hg 18, padding with ±20,000 bases, and gene count range of above 5 and less than 100. For pathway database, we applied the same GO database both in ICSNPathway and GSA-SNP. We applied Fisher Statistics to combine the nominal *P* values from ICSNPathway and GSA-SNP Software to identify pathways that show consistent significance by both methods [22]. To analyze and visualize the pathways identified in the GWAS, GeneMANIA software was used [23]. In GeneMANIA, we chose the options as follows: automatically selected weighting method for network weighting; twenty genes for the number of results to be displayed. The rest of options were set as default in the software.

## Results

### Genome-wide associations with CRP levels and their replication

**Figure 1:**
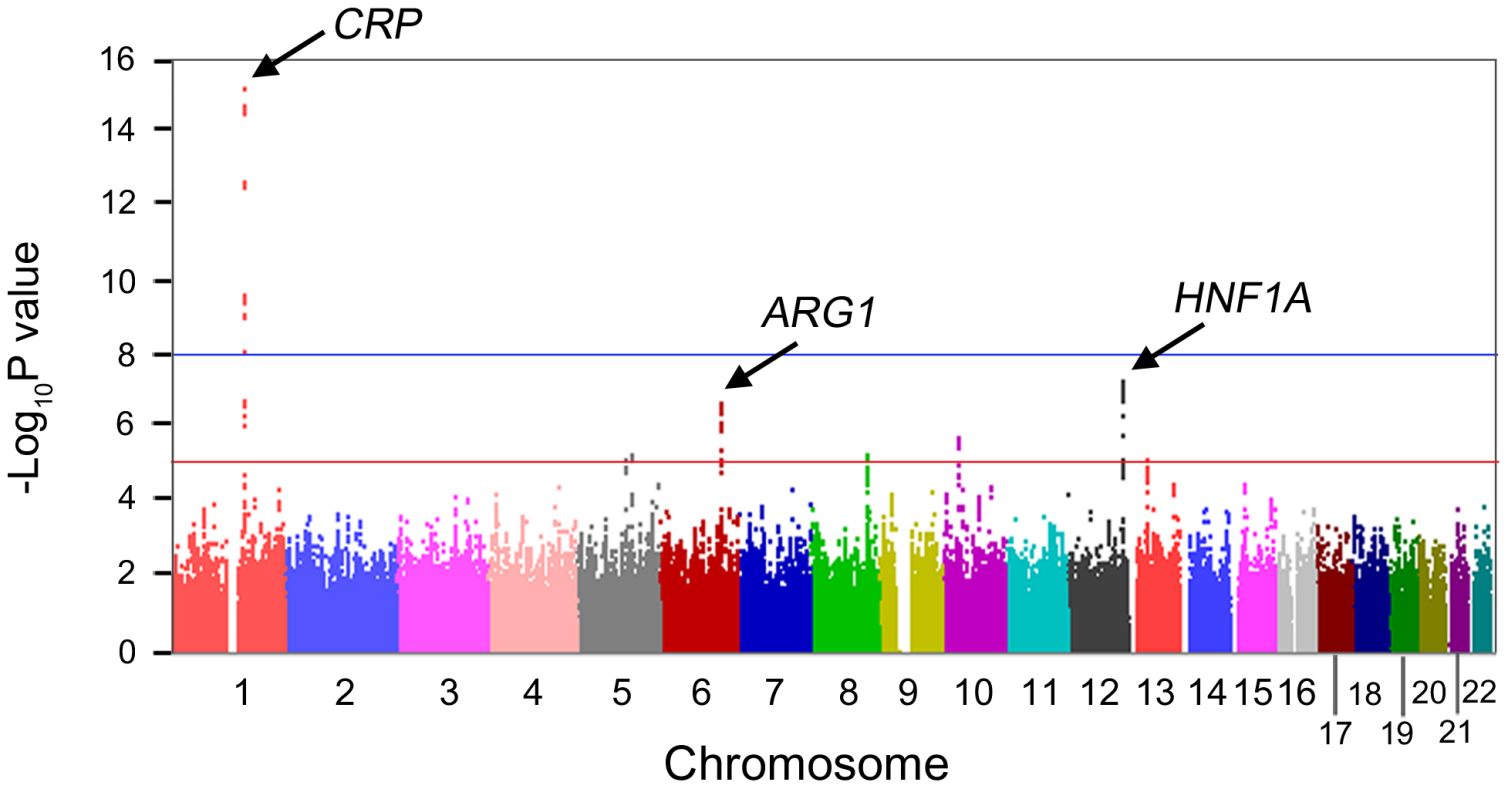
Manhattan plot showing GWAS results for serum CRP levels in 7,626 Korean subjects. The blue horizontal line (*P*<10^−8^) denotes the general threshold for genome-wide significance. The red horizontal line (*P*<10^−5^) denotes the threshold for selecting loci for stage 2 test. The arrow heads indicate three significant loci that passed the threshold.

The general characteristics of stage 1 and stage 2 subjects are summarized in Table S1 in File S1. The stage 1 set consisted of 7,626 unrelated Korean subjects (3,586 men and 4,040 women) and the stage 2 set consisted of 903 Korean individuals (518 male and 385 female). The mean ages of the stage 1 and 2 subjects were 52.5±8.6 and 41.8±8.6 years, respectively. The mean values of HDL, triglycerides and fasting glucose levels of phase 1 participants were 44.9±10.1, 160.2±102.2, and 85.5±15.8, respectively. The GWASs for CRP levels in Korean individuals were performed with imputed SNPs using HapMap II data. The overall results of the GWAS analyses with the additive model are shown as a Manhattan plot (Figure 1) and as a quantile-quantile plot (Figure S1 in File S1). The genomic control inflation factor (λ_GC_) was 1.0, indicating no evidence of type 1 error inflation.

Eighteen SNPs in the *CRP* locus and one SNP in the *HNF1A* locus were below the traditional genome-wide significance criterion (5×10^−8^) (Figure 1 and Table S2 in File S1). We applied a less stringent criterion (*P*<1×10^−5^) to select SNPs for phase 2 study. Ninety-eight SNPs in eight loci passed the threshold and the CRP levels were largely correlated with the genotypes of each SNP (Table S2 in File S1). They included 26 SNPs in the *CRP* locus (1q23.2) (top significant SNP: rs7553007, *P* = 7.34×10^−16^), 17 in the *HNF1A* locus (12q24.31) (top significant SNP rs1169310, *P* = 4.95×10^−8^), 40 in the 6q23.2 locus near *ARG1* (arginase 1) (top significant SNP: rs2608951, *P* = 1.96×10^−7^), two in the *SNCAIP* locus (5q23.2) (top significant SNP rs1841972, *P* = 4.87×10^−6^), one in the *EFNA5* (5q21.3) (SNP rs12517578, *P* = 6.98×10^−6^), five in the *TNFRSF11B* (8q24.12) (top significant SNP rs2062375, *P* = 5.22×10^−6^), five in the *ARHGAP12* (10p11.22) (top significant SNP rs796126, *P* = 1.76×10^−6^), and one in the *TNFSF11* locus (13q14.11) (top significant SNP rs17596685, *P* = 6.69×10^−6^).

To validate the association of the 98 SNPs identified by GWAS, we examined the available 92 SNPs in an independent stage 2 set of 903 Korean individuals (significance criteria of *P*<0.05 for stage 2). Among them, SNPs in the *CRP*, *HNF1A*, and *ARG1* loci were found to be consistently significant (Table S3 in File S1). However, none of the SNPs in the chromosome 5, 8, 10 and 13 loci were significant in the stage 2 set. In a subsequent meta-analysis of the stage 1 and stage 2 results, all three loci were more strongly associated with CRP levels than in stage 1 and reached the traditional genome-wide significance criterion (5×10^−8^): 19 SNPs in the *CRP* locus (1q23.2) (most significant SNP: rs7553007, *P*_meta_ = 1.72×10^−16^), 17 SNPs in the *HNF1A* locus (12q24.31) (most significant SNP: rs2393791, *P*_meta_ = 2.90×10^−10^), and 21 SNPs in the *ARG1* locus (6q23.2) (most significant SNP: rs9375813, *P*_meta_ = 2.85×10^−8^) (Table 1). Details are available in Table S3 in File S1. The results from stage 1, stage 2, and meta-analysis together indicate that the three loci (*CRP* in 1q23.2, *HNF1A* in 12q24.31 and *ARG1* in 6q23.2) were consistently significant (Table S2 and S3 in File S1).

**Table 1:** Results of the genome-wide association study of serum CRP levels.

SNP*	Chr	Position†	Nearest gene	Minor allele	MAF	Stage 1 (n = 7626)		Stage 2 (n = 903)		Meta-analysis (n = 8529)		Q	I^2^
						β (SE)	*P*	β (SE)	*P*	β	*P*		
rs7553007	1	157965173	*CRP*	C	0.369	0.182 (0.023)	7.34×10^−16^	0.099 (0.042)	1.93×10^−2^	0.164	1.72×10^−16^	0.085	66.23
rs2393791	12	119908339	*HNF1A*	G	0.479	−0.118 (0.022)	8.89×10^−8^	−0.138 (0.041)	8.28×10^−4^	−0.122	2.90×10^−10^	0.665	0
rs9375813	6	131798940	*ARG1*	A	0.115	−0.177 (0.034)	2.30×10^−7^	−0.132 (0.063)	3.73×10^−2^	−0.167	2.85×10^−8^	0.538	0

Chr, chromosome; MAF, minor allele frequency; CRP, C-reactive protein; SE, standard error; β, Effect size of a minor allele on natural-log-transformed CRP; Q, p-value for Cochrane's Q statistic assessing if combining studies are homogeneous; I^2^, I-squared index quantifying heterogeneity.

*Most significantly associated SNP in each locus based on the meta analysis results were summarized.

† SNP positions were based on the NCBI human genome build 36.3 (hg18).

**Figure 2:**
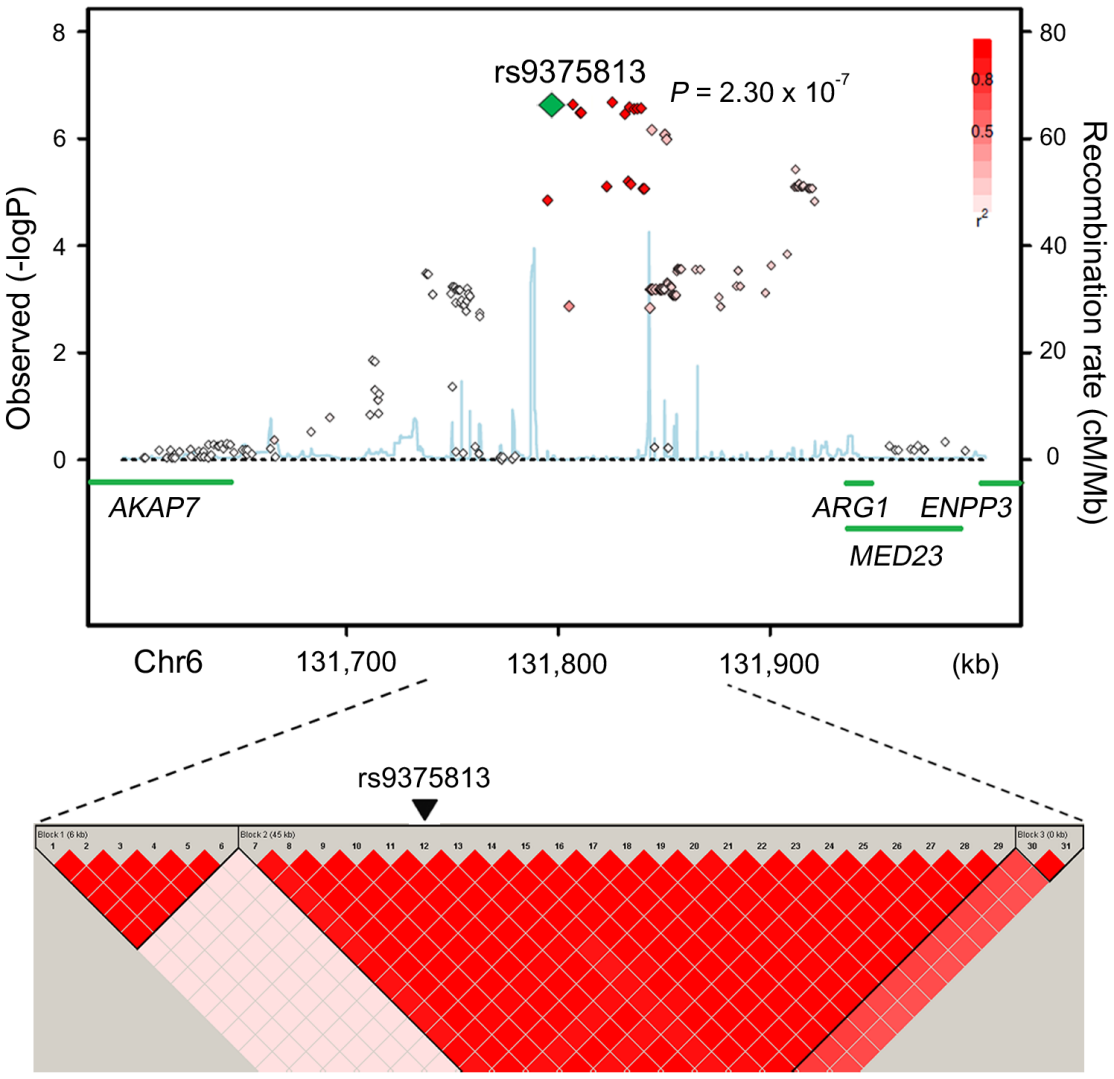
Regional plot of the SNPs in the *ARG1* locus (up) and the LD relationship among these SNPs (down). Data are shown for the *ARG1* locus around rs9375813. Diamond-shaped dots represent -log_10_ (*P*-values) of SNPs, and green diamond in the LD plot indicates the most significant SNP. The strength of LD relationship (*r* ^2^) between the most strongly associated SNP and the other SNPs is presented with red color intensities based on JPT+CHB HapMap data. The light blue curve shows recombination rates drawn based on JPT+CHB HapMap data. Green bars represent the coding genes in this region.

Among these three significant loci, the *CRP* and *HNF1A* loci are known to be associated with CRP levels [4], [24]; however, the *ARG1* locus has not been reported as being associated with CRP levels. In the *ARG1* locus, 22 neighboring SNPs of the rs9375813 cluster in about a 100 kb upstream region of the *ARG1* gene were all in strong LD with one another (Figure 2). The *P*-values and the LD values of the neighboring SNPs are summarized in Table S4 in File S1. Similarly, neighboring SNPs of rs7553007 in the *CRP* locus and those of rs2393791 in the *HNF1A* locus were well clustered with strong LD. The regional associations and LD plots of these loci are shown in Figure S2 in File S1.

### Replication of previously identified SNPs in GWAS

We observed whether the significant loci previously reported as relevant to CRP levels in European and Japanese populations, including *CRP, HNF1A, IL6R, GCKR, IL6*, and *APOE-CI-CII* cluster [4], [6]–[7] were replicated in our study. Six of the nine loci were found to be replicated in our study (Table 2). Details of all the SNPs in the nine loci are available in Table S5 in File S1.

**Table 2:** Association of previously reported CRP-related loci.

SNP*	Chr	Position† (bp)	Nearest Gene	MAF	Type‡	Minor allele	Effect Size	*P*
rs2186245	1	65890652	*LEPR*	0.24	imputed	C	−0.018	0.4948
rs4291477	1	65963663	*PDE4B*	0.17	genotyped	G	−0.029	0.3151
rs8192284	1	152693594	*IL6R*	0.44	imputed	C	−0.059	0.0087
rs7553007	1	157965173	*CRP*	0.37	genotyped	G	0.182	7.34×10^−16^
rs1260333	2	27602128	*GCKR*	0.46	imputed	G	−0.062	0.0054
rs2097677	7	22699364	*IL6*	0.24	imputed	A	0.091	0.0004
rs10778213	12	102019281	*ASCL1*	0.17	imputed	T	0.055	0.0607
rs735396	12	119923227	*HNF1A*	0.44	imputed	T	0.121	5.34×10^−8^
rs4420638	19	50114786	*APOE-CI-CII cluster*	0.11	genotyped	G	−0.098	0.0040

*The most significant SNPs from each locus were shown.

† Position is based on NCBI human genome build 36.3 (hg18).

‡ Type indicates if a SNP is genotyped or imputed. Chr, chromosome; MAF, minor allele frequency.

### SNP prioritization

We performed SNP prioritization analysis to identify SNPs with mediocre p-values, but with potential for high impact using GWASrap tool [19]. Most top rank SNPs still remained significant after SNP prioritization (Table S6 in File S1). However, for three SNPs, rs2608912, rs2608976, rs2608921, their ranking of significance levels became highly elevated after SNP prioritization from 87th, 88th and 81th to 16th, 17th and 19th, respectively. Interestingly, the three SNPs are located about 17∼25 kb upstream of *ARG1* gene and in perfect LD among themselves, and also in moderate LD with rs9375813 (D′ = 0.671) (Figure S3 in File S1).

### Associations of the CRP-associated SNPs with cardiovascular phenotypes

We conducted logistic regression analysis to evaluate the associations between CRP-related SNPs with disease phenotypes such as CHD (n = 65), myocardial infarction (MI; n = 55), and hypertension (n = 1,115). The SNP in *ARG1* (rs9375813) showed a marginal association with hypertension (*P* = 0.0440). The other two SNPs in *CRP* and *HNF1A* did not show any significant associations with any of the traits (Table S7 in File S1).

### Pathway analysis

In addition to GWAS, we performed a pathway analysis to identify more variants and pathways that may influence CRP levels. To minimize the potential bias of any single algorithm, we chose two pathway analysis algorithms to ensure the validity of the identified pathways. We used ICSNPathway software to identify candidate SNPs and mechanisms that contribute to CRP level and to generate pathway hypotheses. In addition, we reconfirmed the pathways using GSA-SNP software.

We used unimputed Phase 1 GWAS *P* values for pathway analysis and identified four candidate SNPs in six pathways (nominal *P*<0.001 and FDR<0.001, Table 3 and Table 4) using ICSNPathway: rs1205 in *CRP,* rs2464196 and rs2464195 in *HNFIA*, and rs1635498 in *EXO1*. Among these, SNPs in the *CRP* and *HNF1A* loci were also identified through GWAS, while a SNP in the *EXO1* gene was exclusively identified through pathway analysis. Among the four variants, three were non-synonymous variants and one was in the regulatory region (Table 3). Although these probes were not present in the Affymetrix SNP 5.0 arrays, all were in strong LD with the SNPs represented in the genotyped data (*r^ 2^* ranged from 0.92 to 1.0).

**Table 3:** Candidate CRP-associated SNPs identified by ICSNPathway analysis.

Candidate SNP	Functional class	Gene	Candidate pathway*	-log_10_(*P*)†	In LD with	*r^2^*	D′	-log_10_(*P*)‡
rs1205	regulatory region	*CRP*	1, 2, 4, 5, 6	-	rs7553007	1	1	15.134
rs1635498	non-synonymous coding (deleterious)	*EXO1*	1, 2,	-	rs1418761	0.92	1	3.079
rs2464196	non-synonymous coding	*HNF1A*	3	-	rs1169313	0.95	1	7.177
rs2464195	non-synonymous coding	*HNF1A*	3	-	rs1169313	1	1	7.177

*The number indicates the index of pathways that are ranked by their statistical significance (FDR) (details in Table 4).

† -log_10_(*P*) in stage 1 phase of the GWAS. The ‘-’ denotes that the SNP was not present in the stage 1 phase of the GWAS.

‡ -log_10_(*P*) for the SNP in the stage 1 phase of the GWAS, which is in LD with the SNP identified by pathway analysis.

**Table 4:** Candidate pathways where CRP-associated SNPs are enriched in both ICSNPathway and GSA-SNP analysis at the <0.001 FDR cut off.

Index*	Candidate pathway	Description	ICSNPathway		GSA-SNP		Top significant genes in ICSNPathway†
			Nominal *P*	FDR	*P*	FDR	
1	GO:0002250	Adaptive immune response	<0.001	<0.001	*-*	*-*	*CRP, EXO1, TLR4, C9, CTSC*
2	GO:0002443	Leukocyte mediated immunity	<0.001	<0.001	*-*	*-*	*CRP, IL6, EXO1, TLR4, C9*
3	GO:0001750	Photoreceptor outer segment	<0.001	<0.001	2.26E-04	<0.001	*HNF1A, RAPGEF4, MAK, RP1, MYRIP*
4	GO:0043498	Cell surface binding	<0.001	<0.001	2.88E-05	<0.001	*CRP, PCSK6, CD36, PRB3, THBS1*
5	GO:0015485	Cholesterol binding	<0.001	<0.001	2.27E-08	<0.001	*CRP, ABCA1, SOAT1, PTCH1, ABCG1*
6	GO:0051635	Bacterial cell surface binding	<0.001	<0.001	9.16E-14	<0.001	*CRP, CD36, PRB3, SFTPD, STAB2*

*Index applicable to ICSNPathway analysis (ranking).

† All significant gene lists are presented in Supplementary Tables S8−S13.

The ‘*-*’ indicates the pathway did not overlap between ICSNPathway and GSA-SAP analysis.

The six pathways identified by ICSNPathway provided six hypothetical biological mechanisms, including the adaptive immune response, leukocyte mediated immunity, photoreceptor outer segment, cell-surface binding, cholesterol binding, and bacterial cell surface binding (Table 4). In addition to the top-ranked four candidate variants in the three genes, other genes such as *TLR4, C9, CD36, ABCG1*, and *ABCA1*, which are known to be related to inflammatory processes, are also involved in these pathways. Detailed information about each pathway is available in Tables S8−S13 in File S1. Of the six pathways identified by ICSNPathway, four overlapped with the pathways defined by GSA-SNP, suggesting the reliability of our pathway analysis (Table 4). Details of the GSA-SNP analysis are available in Table S14 in File S1. When we applied Fisher's method to combine the nominal *P* values of ICSNPathway and GSA-SNP, all four pathways showed consistent significance (*P*<0.001) (Table S15 in File S1).

### Network analysis of identified pathways

To analyze and visualize the pathways identified in GWAS, GeneMANIA network analysis was performed. Several new genes and gene networks were discovered through the analysis of each pathway. Details of each pathway are available in Figures S4−S7 in File S1.

## Discussion

We applied the combined methods of GWAS and pathway analysis to unravel the genetic polymorphisms associated with CRP levels in 8,529 Korean individuals. Although GWAS has become the standard approach for the investigation of associations between common variants and susceptibility to complex diseases [25], a certain amount of biologically meaningful markers and genes can be missed because of the stringent statistical threshold applied to minimize false-positive findings [9]. Pathway analysis can complement the GWAS approach in estimating genetic susceptibility to complex diseases like cardiovascular disease and type-2 diabetes through evaluating the cumulative effects of functionally related genes [26]. By combining GWAS and pathway analysis, we identified both well-known and novel genetic variants associated with CRP levels.

Through independent two-stage GWAS and meta-analysis, three loci (*CRP* in 1q23.2, *HNF1A* in 12q24.31, and *ARG1* in 6q23.2) were found to be consistently significant and satisfied the traditional genome-wide significance criterion (5×10^−8^). Other than these three loci, we also identified variants in the *EFNA5*, *TNFRSF11B*, and *C12orf43* loci. Although none were significant in stage 2 testing nor reached the traditional level of significance in the meta analysis, they are known to be related to the development of CHD [27].

The variants that showed the strongest associations were located in and around the *CRP* locus. This is consistent with previous GWASs that indicated a strong association of variants in the *CRP* and *HNF1A* loci with CRP levels in people of European, Asian, and African American ancestries [4], [6], [28]. The second most significant variant in our study was in the *HNF1A* locus. Recently, Kong *et al.* reported the association of a *HNF1A* polymorphism (rs2393791) with CRP levels and other phenotypes such as arthritis, tuberculosis, and γ-GTP in Korean individuals [29]. HNF1A binds to the *CRP* promoter and is involved in the regulation of CRP [5].

The most notable finding in this study was a significant association of rs9375813 near the *ARG1* gene with CRP levels in the Korean population. This newly identified variant in the 6q23.2 chromosomal region is located approximately 100 kb upstream of *ARG1* and 150 kb downstream of *AKAP7*. The LD block, where rs9375813 is located, extends into *ARG1* but not into *AKAP7*. In addition, three SNPs located about 17∼25 kb upstream of *ARG1* gene were also found to be significantly associated with the CRP level in SNP prioritization analysis and in perfect LD among themselves, and also in moderate LD with rs9375813. All these data suggest that *ARG1* is related to the CRP level. Arginase is one of the enzymes of the urea cycle in the liver and is critically involved in various aspects of inflammation [30]. Although an association between *ARG1* polymorphisms and the level of CRP has not been reported, associations of *ARG1* polymorphism with CVD and asthma have been reported [31], [32]. It is well-known that arginase counteracts nitric oxide (NO) synthase and interferes with beneficial NO-mediated effects, including vasodilation, decreased vascular smooth muscle cell proliferation, decreased interaction between white blood cells and the vascular endothelium, and decreased platelet aggregation [33]. Regarding the relationship between arginase and CRP levels, Bekpinar *et al*. reported that the level of arginase was inversely correlated with that of hsCRP [34]. Moreover, *ARG1* mRNA levels are reported to be positively associated with the up-regulation of soluble intercellular adhesion molecule-1, which is a circulating biomarker for endothelial dysfunction [35]. Combining the results from previous reports along with our data presented here, we hypothesize that *ARG1* polymorphisms or pathways might play a role in CRP level variation and cardiovascular traits.

In this study, *ARG1* polymorphisms, including the A allele of rs9375813, were associated with lower CRP levels. To explore the possibility of whether these *ARG1* polymorphisms may be associated with a lower risk of CVD, we evaluated the association of *ARG1* SNPs with a history of MI, CHD, and hypertension in the discovery subjects. In our logistic regression analysis of the top significant SNPs of *CRP*, *HNF1A,* and *ARG1* loci with cardiovascular phenotypes, the rs9375813 in *ARG1* showed a marginally significant association with hypertension (*P*<0.044), however, the other SNPs did not show any significant associations with any of the traits. This result is in agreement with a report by Elliott et al. that found no association of variants in the *CRP* locus and CVD in a Mendelian randomization study of more than 28,000 cases and 100,000 controls [24]. However, we cannot exclude the possibility that an effect may not be detected due to limitations in CRP measurement itself, including the cross-sectional nature of the measurement and the limited information available on confounding variables such as medication history or the presence of active inflammation at the time of blood sampling. Also, taking the relatively low prevalence of the other phenotypes in our study samples (55 MI and 65 CHD out of 7,626 subjects) into consideration, further analysis with more cases may help to evlauate the association more conclusively between the CRP level-associated SNPs and cardiovascular diseases. In spite of the limitations described above, it's worth noting that our study population is largely disease free population which has already been used in large-scale GWASs of similar traits [36], [10]. Moreover, we have removed the individuals with diabetes mellitus who may have increased level of inflammation [37], [38] from our study. The rs9375813 MAF varies widely between ethnicities: 0.09 in Europeans (HapMap CEPH), 0.20 in Africans (HapMap YRI), and 0.15 in Asians (HapMap HCB and JPT). Considering that most of the large-scale GWASs for CRP levels have been performed in Europeans [4], [5], the relatively lower MAF in European people might be one of the reasons why this locus has not been identified in earlier studies. Asians and Africans seem to have relatively higher MAFs than Europeans, but the LD structures are very different between them (Figure S3 in File S1). At the present time, it remains unclear whether the association between rs9375813 and CRP levels is Asian-specific; further studies in diverse ethnic groups will be required to clarify this issue.

In this study, six of the nine significant loci reported in previous GWASs on CRP levels in diverse ethnic groups were replicated in our Korean population study (Table 2). This result suggests that these SNPs may be universally linked to CRP levels in human beings. Among the replicated SNP loci, those in the *IL6R* and *IL6* genes showed the same directional effect with ours [4], [6] while those in *GCKR* and *HNF1A* presented the opposite directional effect to ours [4]. Interestingly, the directional effect of rs7553007 in *CRP* differed between studies: A study with Hispanic American individuals showed similar direction results as presented here [7], but other studies with Europeans and West Africans showed the opposite direction [4], [28]. In addition, rs10778213 in the *ASCL1* gene, which was identified in American women, was not found in a Japanese population (*P* = 0.54) nor in this study (*P* = 0.06).

To identify more reliable pathways and minimize false positive findings, we used two different software packages, ICSNPathway and GSA-SNP. In both softwares, using a full list of GWAS SNP *P*-values is desirable. ICSNPathway selects the best –log *P* value, while in GSA-SNP, user has the option of selecting the best or second best SNP within a gene boundary to be assign to the gene. ICSNPathway compares the distribution of the member gene scores of a gene set to all the genes using Kolmogor-Smirnov like running-sum statistics. Variation of the number of member genes among gene sets is taken care of by multiplying it to factor m1/m2, where m1 is the proportion of significant genes defined as genes mapped with at least one of the top 5% most significant SNPs of all SNPs in GWAS for pathways and m2 is the proportion of significant genes for all the genes in the GWAS [20]. On the other hand, in GSA-SNP, the scores of its member genes are averaged for each gene set and significance is estimated using Z-statistics of these scores [22]. FDR is computed for multiple testing corrections.

Four out of six pathways from ICSNPathway analysis overlapped with pathways identified by GSA-SNP at the FDR cut off of <0.001 (Table 4), indicating reliability of the identified pathways. When we applied Fisher Statistics to combine the nominal *P* values of ICSNPathway and GSA-SNP, all four pathways showed consistent significance (*P*<0.001). However, a number of the pathways were identified only by GSA-SNP which suggests that there is a possibility of false positives in the pathway analysis results and cross validation will help to rule out them. The significantly enriched genes in these pathways, such as *CRP, HNF1A*, *PCSK6, CD36*, and *ABCA1*, have a link either to inflammation or CVD. The top significant SNP in this study, CRP, has also been reported as a top significant gene associated with CRP levels in almost all GWASs reported so far [4], [7]. In addition to *CRP*, *CD36* is one of the key genes enriched in the cell surface binding pathway, and has been reported to be associated with inflammation-mediated diseases such as atherosclerosis [39]. A number of studies have suggested a plausible mechanism that may link genes enriched in the cholesterol binding pathway to inflammation or to the etiology of atherosclerosis [40]. Indeed, the key genes enriched in the cholesterol binding pathway have links to cholesterol efflux (*ABCG1*), inflammation, and atherosclerosis (*ABCB1* and *APOA2*) [41]. These results strongly suggest that the genes enriched in the cholesterol binding and cell surface binding pathways are involved in the regulation of inflammation, which is linked to CRP levels and may be involved in atherosclerosis pathogenesis. Although the term photoreceptor outer segment has no biological relevance to CRP or inflammation, the genes enriched in this pathway have biological relevance to CRP (*HNF1A*) [5], high triglycerides (*PCDH15*) [42] and metabolic syndrome (*GNAT3*) [43]. Likewise, the genes enriched in bacterial cell surface binding pathway have relevance to the CRP level (*CRP*), atherosclerosis (*CD36*) and inflammation (*PCSK6*) [44]–[46]. Two pathways identified by ICSNPathway, the adaptive immune response and leukocyte mediated immunity pathways, did not overlap with pathways identified by GSA-SNP. Although not overlapped in both software packages, genes enriched in the two pathways have a putative connection with inflammation and CVDs [47], [48]. In addition, some pathways identified only by GSA-SNP have potential implications in the inflammation and CVD pathways. GSA-SNP identified 116 pathways at the FDR cutoff of <0.001 in this study (Table S14 in File S1), with the top-ranked pathway identified as low-density lipoprotein particle binding. The top enriched genes in this pathway were *CRP, CDH13, STAB2, THBS1*, and *SORL1. CDH13* variants have been reported to be associated with hypertension [49]. It is worth noting that arginase II was enriched in cellular response to the interferon gamma pathway in GSA-SNP analysis (Table S12 in File S1). Although arginase I and arginase II are localized differently, both isoforms catalyze the hydrolysis of L-arginine to L-ornithine and urea [50]. This result is additional supporting evidence that *ARG1* polymorphism or pathways related with *ARG1* might play a role in CRP level variation and cardiovascular traits.

To analyze and visualize the pathways identified in the GWAS, GeneMANIA network analysis was performed. This analysis can help to find new genes that have phenotypic relations with query gene, which will be useful for selecting the candidates for further functional study. We also examined whether the enriched genes in the network analysis were reported as being associated with immune response or CVD. In the photoreceptor outer segment pathway, some of the newly identified genes such as *RAP1A* and *CACNA1C* have a connection with inflammation [51], [52]. Interestingly, *CACNA1C* has reported to be enriched in pathway analysis of inflammatory conditions such as Crohn's disease [51], [52]. Among the newly identified genes in that pathway, *PDE6H* and *MYL6B* had higher weight in the network (Figure S4 in File S1). Some of the newly identified genes in the cholesterol binding pathway, *SHH*, *APOF* and *APOC1*, were also associated with cholesterol transport or CVD [53], [54]. Among them, *DHH*, *SHH* and *PTCH2* had higher weight in the network (Figure S6 in File S1). The key members of the cell surface binding and bacterial cell surface binding networks such as *TLR6*, *CD14*, *CD244*, *CD58*, *SCARB2*, and *SCARB1* have interconnected roles in inflammatory responses and CVD [55], [56]. Among them, *SCARB1* and *SCARB2* had higher weight in the bacterial cell surface binding network (Figure S7 in File S1). Especially, *SCARB1* plays a vital role in reverse cholesterol transport and is also involved in the removal of cholesterol [57]. Taken together, through GeneMANIA network analysis of the identified pathways, we were able to identify more genes related to inflammation or CVD.

Overall, some CRP-associated polymorphisms enriched in the pathways are involved in the inflammation or pathogenesis of CVDs. Although we did not explore the biological effects of polymorphisms identified through pathway analysis, these genes and pathways may help to generate hypotheses for further functional studies investigating the inter-individual differences in CRP levels and CVD risk.

Our study has several limitations. First, the nature of serum CRP as an acute reactant itself can be a limitation. The baseline CRP level in each individual should be estimated based on repetitive measurements, preferably in the absence of acute inflammatory conditions, which we could not achieve in this study. Second, the sample size of the replication set may not be large enough to verify the potential associations with CRP levels. To confirm the associations we identified, replication in a large meta-analysis of CRP GWAS specifically in Asian population will be required. After that, meaningful functional studies on the SNP shoud follow.

In conclusion, by combining GWAS, pathway and gene network analysis, we identified novel *ARG1* variants and a number of interesting candidate genes related to inflammatory processes and CVDs such as *CRP, HNF1A*, *PCSK6, CD36*, and *ABCA1* in the Korean population. Our results also strongly corroborate the previously reported loci (*CRP, HNF1A, IL6)* known to be associated with CRP levels in diverse ethnic groups. This study highlights the effectiveness of combining GWAS and pathway analysis in identifying new genetic variants in meaningful pathways, which can improve our understanding of the genetic mechanisms behind variations in CRP levels.

## Supporting Information

File S1 **Figure S1**, A Quantile–Quantile plot of *P*-values in the GWAS for serum CRP levels (Stage 1). The horizontal axis indicates the expected -log_10_ (*P*-values). The vertical axis indicates the observed -log_10_ (*P*-values). The red line represents y = x. **Figure S2**, (A) Stage 1 data showing a regional association (upper panel) and linkage disequilibrium (LD; lower panel) plots of the *CRP* locus around rs7553007. Arrow head represents rs7553007. (B) Stage 1 data showing a regional association (upper panel) and linkage disequilibrium (LD; lower panel) plots of the *HNF1A* locus around rs2393791. Arrow head represents rs2393791. **Figure S3**, Pairwise linkage disequilibrium (LD) between the selected SNPs in *ARG1* locus around rs9375813. LD plots for Korea population were drawn using the genotype data from the present study, whereas LD plots for Japanese, Chinese, Europeans and Africans were made from genotype data from HapMap Stage 2. Blue ID indicates the most significant SNP. Purple ID indicates the SNPs whose rank got elevated after re-prioritization. **Figure S4**, Gene network of photoreceptor outer segment pathway by GeneMANIA analysis. Using the genes identified from pathway analysis, GeneMANIA network analysis was performed. Query genes are depicted as black nodes and discovered genes are depicted as gray nodes. Edges show different interactions among genes; purple indicates for co-expression; light-blue indicates for pathway; dark yellow indicates for shared protein domains; red indicates for physical interactions; dark blue indicates for co-localization; green indicates for genetic interactions. Node sizes are determined according to their weight in the network. **Figure S5**, Gene network of cell surface binding pathway by GeneMANIA. **Figure S6**, Gene network of cholesterol binding pathway by GeneMANIA. **Figure S7**, Gene network of bacterial cell surface binding pathway by GeneMANIA. **Table S1**, Characteristics of the subjects in stage 1 and 2 data. The stage 1 consists of 7,626 unrelated Korean subjects (3,586 men and 4,040 women) and the stage 2 consists of 903 Korean individuals (518 male and 385 female). **Table S2**, SNP loci associated with serum CRP levels in the stage 1 data. **Table S3**, SNP loci associated with serum CRP levels in the stage 2 data and meta-analysis. **Table S4**, *P*-values and LD values of neighboring SNPs of the rs9375813 in the *ARG1* locus. **Table S5**, Associations of the previously reported CRP-related loci. Based on genotyped and imputed SNP data, we observed the associations of previously reported CRP-associated loci. **Table S6**, Re-prioritized genetic variants list after GWAS. **Table S7**, Associations of the top significant SNP polymorphisms on cardiovascular disease traits based on the whole KARE samples (7,626 samples). **Table S8**, Genes mapped with variant in adaptive immune response. We performed a pathway analysis to identify more variants and pathways that may influence CRP levels using ICSNPathway software and reconfirmed the identified pathways with GSA-SNP software. This table shows the genes mapped with variants in adaptive immune response among the ten candidate pathways enriched with CRP-associated SNPs in ICSNPathay analysis. **Table S9**, Genes mapped with variant in leukocyte mediated immunity. **Table S10**, Genes mapped with variant in photoreceptor outer segment. **Table S11**, Genes mapped with variant in cell surface binding. **Table S12**, Genes mapped with variant in cholesterol binding. **Table S13**, Genes mapped with variant in bacterial cell surface binding. **Table S14**, Pathway analysis results of GSA-SNP software. **Table S15**, Combined *P* value estimation of ICSNPathway and GSA-SNP by Fisher Statistics.
(PDF)
