# Supplementary material for: New Variants Including ARG1 Polymorphisms Associated with C-Reactive Protein Levels Identified by Genome-Wide Association and Pathway Analysis
Source: PLoS One. 2014 Apr 24;9(4):e95866. doi: 10.1371/journal.pone.0095866 (PMC3999194; doi:10.1371/journal.pone.0095866)
Supplement: File S1 — Figure S1, A Quantile–Quantile plot of P-values in the GWAS for serum CRP levels (Stage 1). The horizontal axis indicates the expected -log10 (P-values). The vertical axis indicates the observed -log10 (P-values). The red line represents y = x. Figure S2, (A) Stage 1 data showing a regional association (upper panel) and linkage disequilibrium (LD; lower panel) plots of the CRP locus around rs7553007. Arrow head represents rs7553007. (B) Stage 1 data showing a regional association (upper panel) and linkage disequilibrium (LD; lower panel) plots of the HNF1A locus around rs2393791. Arrow head represents rs2393791. Figure S3, Pairwise linkage disequilibrium (LD) between the selected SNPs in ARG1 locus around rs9375813. LD plots for Korea population were drawn using the genotype data from the present study, whereas LD plots for Japanese, Chinese, Europeans and Africans were made from genotype data from HapMap Stage 2. Blue ID indicates the most significant SNP. Purple ID indicates the SNPs whose rank got elevated after re-prioritization. Figure S4, Gene network of photoreceptor outer segment pathway by GeneMANIA analysis. Using the genes identified from pathway analysis, GeneMANIA network analysis was performed. Query genes are depicted as black nodes and discovered genes are depicted as gray nodes. Edges show different interactions among genes; purple indicates for co-expression; light-blue indicates for pathway; dark yellow indicates for shared protein domains; red indicates for physical interactions; dark blue indicates for co-localization; green indicates for genetic interactions. Node sizes are determined according to their weight in the network. Figure S5, Gene network of cell surface binding pathway by GeneMANIA. Figure S6, Gene network of cholesterol binding pathway by GeneMANIA. Figure S7, Gene network of bacterial cell surface binding pathway by GeneMANIA. Table S1, Characteristics of the subjects in stage 1 and 2 data. The stage 1 consists of 7,626 unrelated [file pone.0095866.s001.pdf]

Supplementary Data

New Variants including *ARG1* polymorphisms associated with C-reactive protein levels identified by genome-wide association and pathway analysis

Nadimuthu Vinayagamoorthy[1,2], Hae-Jin Hu[1,2], Seon-Hee Yim[1,3], Seung-Hyun Jung[1,2], Jaeseong Jo[4], Sun Ha Jee[4] and Yeun-Jun Chung[1,2,*]

[1]Integrated Research Center for Genome Polymorphism, [2]Department of Microbiology, [3]Department of Medical Education, The Catholic University of Korea, College of Medicine, 222 Banpo-daero, Seocho-gu, Seoul, 137-701, Korea; [4]Institute of Health Promotion, Graduate School of Public Health, Yonsei University, Seoul, 120-749, Korea

Supplementary Tables

Table S1. Characteristics of the subjects in stage 1 and 2 data.

	**Stage 1**	**Stage 2**
N	7626	903
Age (years)*	52.5±8.6	41.8±8.6
Male (%)	3586(47.0)	518 (57.4)
BMI (kg/m^2)*	24.6±3.1	23.7±3.2
Smoker (%)	25.6	45.1
Drinker (%)	53.3	82.9
Pulse rate*	63.6±7.4	65.1±9.6
CRP(mg/dL)*	0.2±0.45	0.1±0.12

*Mean ± SD

Table S2. SNP loci associated with serum CRP levels in the stage 1 data.

SNP*	Locus	Nearest Gene	Position**	Minor allele	MAF	Median CRP level (mg/dL)			Type***	Effect Size	SE	P
						11	12	22				
rs7553007	1q23.2	*CRP*	157965173	G	0.369	0.17	0.15	0.13	genotyped	0.182	0.023	7.34E-16
rs1205	1q23.2	*CRP*	157948857	C	0.364	0.17	0.15	0.13	imputed	0.181	0.023	2.21E-15
rs2808629	1q23.2	*CRP*	157943420	G	0.363	0.17	0.15	0.13	imputed	0.181	0.023	2.50E-15
rs2794520	1q23.2	*CRP*	157945440	C	0.364	0.17	0.15	0.13	imputed	0.181	0.023	2.82E-15
rs2808628	1q23.2	*CRP*	157942635	G	0.360	0.17	0.15	0.13	imputed	0.182	0.023	3.43E-15
rs876537	1q23.2	*CRP*	157941557	C	0.359	0.17	0.15	0.13	imputed	0.182	0.023	3.61E-15
rs2027471	1q23.2	*CRP*	157956012	T	0.344	0.17	0.15	0.13	imputed	0.171	0.023	2.30E-13
rs1341665	1q23.2	*CRP*	157958183	G	0.344	0.17	0.15	0.13	imputed	0.17	0.023	2.98E-13
rs16842599	1q23.2	*CRP*	157964099	C	0.140	0.18	0.16	0.13	imputed	0.199	0.031	2.39E-10
rs16842559	1q23.2	*CRP*	157942795	C	0.140	0.18	0.16	0.13	imputed	0.199	0.031	2.54E-10
rs16842568	1q23.2	*CRP*	157942844	G	0.140	0.18	0.16	0.13	imputed	0.199	0.031	2.54E-10
rs12081480	1q23.2	*CRP*	157973614	G	0.140	0.18	0.16	0.13	imputed	0.199	0.032	2.96E-10
rs12081252	1q23.2	*CRP*	157973137	C	0.140	0.18	0.16	0.13	imputed	0.198	0.031	3.28E-10
rs12068753	1q23.2	*CRP*	157959161	A	0.140	0.18	0.16	0.13	genotyped	0.197	0.031	3.41E-10
rs12081264	1q23.2	*CRP*	157973184	C	0.140	0.18	0.16	0.13	imputed	0.197	0.031	3.81E-10
rs3093075	1q23.2	*CRP*	157946537	T	0.145	0.19	0.16	0.13	imputed	0.190	0.031	8.66E-10
rs3093077	1q23.2	*CRP*	157946260	C	0.145	0.19	0.16	0.13	genotyped	0.190	0.031	9.30E-10
rs16842502	1q23.2	*CRP*	157920487	A	0.140	0.18	0.16	0.13	imputed	0.184	0.032	7.80E-09
rs2794526	1q23.2	*CRP*	157892102	A	0.125	0.18	0.16	0.14	imputed	0.172	0.033	1.79E-07

rs4275453	1q23.2	*CRP*	157974671	C	0.255	0.17	0.15	0.13	imputed	0.132	0.025	2.06E-07
rs1446976	1q23.2	*CRP*	157895818	G	0.125	0.18	0.16	0.14	imputed	0.170	0.033	2.32E-07
rs1446975	1q23.2	*CRP*	157895966	C	0.125	0.18	0.16	0.14	imputed	0.170	0.033	2.32E-07
rs1891186	1q23.2	*CRP*	157895645	G	0.126	0.18	0.16	0.13	imputed	0.170	0.033	2.34E-07
rs1891187	1q23.2	*CRP*	157895677	A	0.125	0.18	0.16	0.13	imputed	0.170	0.033	2.34E-07
rs12087465	1q23.2	*CRP*	157891923	A	0.109	0.17	0.16	0.14	imputed	0.178	0.035	4.17E-07
rs2794500	1q23.2	*CRP*	157901645	T	0.125	0.18	0.16	0.13	genotyped	0.162	0.033	7.94E-07
rs1841972	5q23.2	*SNCAIP*	121824037	G	0.226	0.15	0.14	0.14	genotyped	0.121	0.026	4.87E-06
rs10478555	5q23.2	*SNCAIP*	121809430	T	0.222	0.15	0.14	0.14	imputed	0.121	0.027	6.78E-06
rs12517578	5q21.3	*EFNA5*	106008730	G	0.355	0.12	0.14	0.14	genotyped	-0.102	0.023	6.98E-06
rs2608951	6q23.2	*ARG1*	131825473	G	0.120	0.13	0.13	0.14	genotyped	-0.174	0.033	1.96E-07
rs9483280	6q23.2	*ARG1*	131798329	G	0.115	0.13	0.13	0.14	imputed	-0.177	0.034	2.02E-07
rs9402328	6q23.2	*ARG1*	131798744	C	0.115	0.13	0.13	0.14	imputed	-0.177	0.034	2.02E-07
rs2143779	6q23.2	*ARG1*	131800478	T	0.115	0.13	0.13	0.14	imputed	-0.177	0.034	2.05E-07
rs9492976	6q23.2	*ARG1*	131797691	A	0.115	0.13	0.13	0.14	imputed	-0.177	0.034	2.06E-07
rs2179771	6q23.2	*ARG1*	131796623	C	0.115	0.13	0.13	0.14	imputed	-0.177	0.034	2.14E-07
rs9375813	6q23.2	*ARG1*	131798940	A	0.115	0.13	0.13	0.14	genotyped	-0.177	0.034	2.30E-07
rs9483281	6q23.2	*ARG1*	131806872	A	0.115	0.13	0.13	0.14	imputed	-0.176	0.034	2.31E-07
rs2608928	6q23.2	*ARG1*	131833564	A	0.119	0.13	0.13	0.14	imputed	-0.173	0.034	2.54E-07
rs2491204	6q23.2	*ARG1*	131839097	T	0.119	0.13	0.13	0.14	imputed	-0.173	0.034	2.69E-07
rs9321294	6q23.2	*ARG1*	131837250	T	0.119	0.13	0.13	0.14	imputed	-0.173	0.034	2.75E-07
rs2608927	6q23.2	*ARG1*	131835736	T	0.119	0.13	0.13	0.14	imputed	-0.172	0.034	2.80E-07

rs6904726	6q23.2	*ARG1*	131810390	A	0.115	0.13	0.13	0.14	imputed	-0.174	0.034	3.26E-07
rs6904733	6q23.2	*ARG1*	131810402	T	0.115	0.13	0.13	0.14	imputed	-0.174	0.034	3.26E-07
rs6927792	6q23.2	*ARG1*	131810800	C	0.115	0.13	0.13	0.14	imputed	-0.174	0.034	3.26E-07
rs2608935	6q23.2	*ARG1*	131831429	A	0.117	0.13	0.13	0.14	genotyped	-0.171	0.034	3.68E-07
rs9492992	6q23.2	*ARG1*	131844120	T	0.151	0.13	0.13	0.14	genotyped	-0.15	0.03	6.88E-07
rs9493000	6q23.2	*ARG1*	131850284	C	0.150	0.13	0.13	0.14	imputed	-0.149	0.03	8.41E-07
rs7740929	6q23.2	*ARG1*	131850315	G	0.150	0.13	0.13	0.14	imputed	-0.149	0.03	8.41E-07
rs3861462	6q23.2	*ARG1*	131851129	C	0.150	0.13	0.13	0.14	imputed	-0.148	0.03	1.04E-06
rs2608913	6q23.2	*ARG1*	131911954	C	0.309	0.14	0.13	0.14	imputed	-0.11	0.024	3.76E-06
rs2807272	6q23.2	*ARG1*	131832938	T	0.109	0.14	0.13	0.14	imputed	-0.157	0.035	6.30E-06
rs2608917	6q23.2	*ARG1*	131913649	G	0.312	0.14	0.13	0.14	imputed	-0.107	0.024	6.93E-06
rs2807273	6q23.2	*ARG1*	131834214	G	0.109	0.14	0.13	0.14	imputed	-0.156	0.035	7.10E-06
rs2446213	6q23.2	*ARG1*	131915724	C	0.312	0.14	0.14	0.14	genotyped	-0.106	0.024	7.55E-06
rs2491209	6q23.2	*ARG1*	131822823	A	0.110	0.14	0.13	0.14	imputed	-0.158	0.035	7.82E-06
rs2608919	6q23.2	*ARG1*	131915028	T	0.312	0.14	0.14	0.14	imputed	-0.106	0.024	7.89E-06
rs2608921	6q23.2	*ARG1*	131915428	T	0.312	0.14	0.14	0.14	imputed	-0.106	0.024	7.89E-06
rs2608915	6q23.2	*ARG1*	131913007	G	0.312	0.14	0.14	0.14	imputed	-0.106	0.024	7.93E-06
rs2608916	6q23.2	*ARG1*	131913298	G	0.312	0.14	0.14	0.14	imputed	-0.106	0.024	7.93E-06
rs2608918	6q23.2	*ARG1*	131914585	T	0.312	0.14	0.14	0.14	imputed	-0.106	0.024	7.93E-06
rs2608912	6q23.2	*ARG1*	131911476	C	0.312	0.14	0.14	0.14	genotyped	-0.106	0.024	7.94E-06
rs2608914	6q23.2	*ARG1*	131912304	A	0.312	0.14	0.14	0.14	imputed	-0.106	0.024	7.94E-06
rs2608982	6q23.2	*ARG1*	131917893	T	0.312	0.14	0.14	0.14	imputed	-0.105	0.024	8.52E-06

rs2781650	6q23.2	*ARG1*	131918414	G	0.312	0.14	0.14	0.14	imputed	-0.105	0.024	8.52E-06
rs2781651	6q23.2	*ARG1*	131918636	G	0.312	0.14	0.14	0.14	imputed	-0.105	0.024	8.52E-06
rs2749933	6q23.2	*ARG1*	131918926	T	0.312	0.14	0.14	0.14	imputed	-0.105	0.024	8.52E-06
rs2608976	6q23.2	*ARG1*	131919868	C	0.312	0.14	0.14	0.14	imputed	-0.105	0.024	8.52E-06
rs4443525	6q23.2	*ARG1*	131839960	A	0.109	0.14	0.13	0.14	imputed	-0.155	0.035	8.64E-06
rs2608940	6q23.2	*ARG1*	131840624	T	0.109	0.14	0.13	0.14	imputed	-0.155	0.035	8.64E-06
rs2062375	8q24.12	*TNFRSF11B*	120046973	G	0.271	0.16	0.15	0.13	imputed	0.113	0.025	5.22E-06
rs9969672	8q24.12	*TNFRSF11B*	120075261	T	0.268	0.15	0.15	0.13	imputed	0.113	0.025	6.29E-06
rs7014574	8q24.12	*TNFRSF11B*	120046258	C	0.268	0.15	0.15	0.13	genotyped	0.111	0.025	6.78E-06
rs11992136	8q24.12	*TNFRSF11B*	120072908	C	0.268	0.15	0.15	0.13	imputed	0.113	0.025	6.92E-06
rs2062377	8q24.12	*TNFRSF11B*	120076601	T	0.271	0.15	0.15	0.13	imputed	0.11	0.025	9.85E-06
rs796126	10p11.22	*ARHGAP12*	32008765	T	0.364	0.13	0.14	0.15	genotyped	-0.109	0.023	1.76E-06
rs796127	10p11.22	*ARHGAP12*	32008735	T	0.364	0.13	0.14	0.15	genotyped	-0.109	0.023	1.95E-06
rs2488384	10p11.22	*ARHGAP12*	32013773	T	0.371	0.13	0.14	0.15	imputed	-0.11	0.023	2.20E-06
rs1775437	10p11.22	*ARHGAP12*	32004232	T	0.359	0.13	0.14	0.15	imputed	-0.108	0.023	2.35E-06
rs796130	10p11.22	*ARHGAP12*	32006513	G	0.360	0.13	0.14	0.15	genotyped	-0.107	0.023	2.97E-06
rs796082	10p11.22	*ZEB1*	31960297	G	0.360	0.13	0.14	0.15	imputed	-0.102	0.023	9.24E-06
rs1169310	12q24.31	*HNF1A*	119923816	G	0.443	0.16	0.14	0.13	imputed	0.121	0.022	4.95E-08
rs1169306	12q24.31	*HNF1A*	119922694	C	0.444	0.16	0.14	0.13	imputed	0.121	0.022	5.15E-08
rs735396	12q24.31	*HNF1A*	119923227	T	0.444	0.16	0.14	0.13	imputed	0.121	0.022	5.34E-08
rs2464195	12q24.31	*HNF1A*	119919858	G	0.444	0.16	0.14	0.13	imputed	0.121	0.022	5.83E-08
rs1169313	12q24.31	*C12orf43*	119927053	T	0.446	0.16	0.14	0.13	genotyped	0.119	0.022	6.66E-08

rs2259816	12q24.31	*HNF1A*	119919970	G	0.446	0.16	0.14	0.13	genotyped	0.119	0.022	7.10E-08
rs2259820	12q24.31	*HNF1A*	119919725	C	0.446	0.16	0.14	0.13	imputed	0.119	0.022	8.58E-08
rs2464196	12q24.31	*HNF1A*	119919810	G	0.446	0.16	0.14	0.13	imputed	0.119	0.022	8.58E-08
rs2393791	12q24.31	*HNF1A*	119908339	C	0.480	0.12	0.14	0.16	genotyped	-0.118	0.022	8.89E-08
rs7310409	12q24.31	*HNF1A*	119909244	A	0.479	0.12	0.14	0.16	imputed	-0.116	0.022	1.38E-07
rs2393775	12q24.31	*HNF1A*	119908957	G	0.482	0.12	0.14	0.16	imputed	-0.116	0.022	1.75E-07
rs1182933	12q24.31	*C12orf43*	119939005	C	0.452	0.16	0.14	0.13	imputed	0.114	0.022	4.05E-07
rs2708101	12q24.31	*OASL*	119972015	C	0.482	0.16	0.14	0.13	genotyped	0.106	0.022	1.36E-06
rs1920792	12q24.31	*HNF1A*	119888967	C	0.468	0.16	0.14	0.13	imputed	0.099	0.022	6.35E-06
rs7305618	12q24.31	*HNF1A*	119887315	T	0.468	0.16	0.14	0.13	imputed	0.099	0.022	6.86E-06
rs1169303	12q24.31	*HNF1A*	119920759	A	0.245	0.17	0.14	0.13	imputed	0.114	0.026	9.47E-06
rs7953249	12q24.31	*HNF1A*	119888107	A	0.469	0.16	0.14	0.13	genotyped	0.097	0.022	9.85E-06
rs17596685	13q14.11	*TNFSF11*	42015201	T	0.352	0.13	0.14	0.14	genotyped	-0.102	0.023	6.69E-06

*98 SNP loci with unadusted *P* value < 10^{-5} were presented; **Positions of the SNPs were based on NCBI human genome build 36.3 (hg18);

*** Type indicates if a SNP is genotype or imputed; Chr, chromosome; MAF, minor allele frequency; CRP, C-reactive protein; SE, standard error;

Table S3. SNP loci associated with serum CRP levels in the stage 2 data and meta-analysis.

SNP*	Nearest gene	Locus	Position	Minor allele	Stage 2		Meta-analysis		Q	I^2
					β (SE)	*P*	β	*P*		
rs7553007	*CRP*	1q23.2	157965173	G	0.099 (0.042)	1.93E-02	0.164	1.72E-16	0.085	66.23
rs1205	*CRP*	1q23.2	157948857	C	0.099 (0.043)	2.07E-02	0.163	5.09E-16	0.093	64.63
rs2808629	*CRP*	1q23.2	157943420	G	0.098 (0.043)	2.30E-02	0.163	6.64E-16	0.088	65.72
rs2794520	*CRP*	1q23.2	157945440	C	0.098 (0.043)	2.30E-02	0.162	7.39E-16	0.089	65.52
rs2808628	*CRP*	1q23.2	157942635	G	0.100 (0.043)	2.00E-02	0.164	7.50E-16	0.097	63.73
rs876537	*CRP*	1q23.2	157941557	C	0.094 (0.043)	3.17E-02	0.163	1.49E-15	0.072	69.15
rs1341665	*CRP*	1q23.2	157958183	G	0.103 (0.043)	1.72E-02	0.155	3.76E-14	0.176	45.42
rs2027471	*CRP*	1q23.2	157956012	T	0.100 (0.043)	2.12E-02	0.155	3.92E-14	0.151	51.52
rs16842599	*CRP*	1q23.2	157964099	C	0.182 (0.058)	1.78E-03	0.195	1.55E-12	0.791	0
rs12081480	*CRP*	1q23.2	157973614	G	0.182 (0.058)	1.80E-03	0.195	1.90E-12	0.795	0
rs16842559	*CRP*	1q23.2	157942795	C	0.179 (0.058)	2.17E-03	0.194	1.99E-12	0.758	0
rs16842568	*CRP*	1q23.2	157942844	G	0.179 (0.058)	2.17E-03	0.194	1.99E-12	0.758	0
rs12081252	*CRP*	1q23.2	157973137	C	0.182 (0.058)	1.78E-03	0.194	2.10E-12	0.805	0
rs12068753	*CRP*	1q23.2	157959161	A	0.182 (0.058)	1.78E-03	0.194	2.18E-12	0.813	0
rs12081264	*CRP*	1q23.2	157973184	C	0.182 (0.058)	1.78E-03	0.194	2.42E-12	0.813	0
rs3093075	*CRP*	1q23.2	157946537	T	0.167 (0.058)	3.76E-03	0.185	1.15E-11	0.728	0
rs3093077	*CRP*	1q23.2	157946260	C	0.168 (0.057)	3.57E-03	0.185	1.18E-11	0.736	0
rs16842502	*CRP*	1q23.2	157920487	A	0.171 (0.058)	3.57E-03	0.181	9.62E-11	0.841	0
rs4275453	*CRP*	1q23.2	157974671	C	0.124 (0.046)	7.90E-03	0.130	5.26E-09	0.871	0
rs12517578	*EFNA5*	5q21.3	106008730	G	-0.005 (0.044)	9.10E-01	-0.082	5.21E-05	0.048	74.34
rs1841972	*SNCAIP*	5q23.2	121824037	G	-0.025 (0.049)	6.04E-01	0.088	1.61E-04	0.008	85.63
rs10478555	*SNCAIP*	5q23.2	121809430	T	-0.021 (0.049)	6.76E-01	0.088	1.77E-04	0.011	84.39

rs9375813	*ARG1*	6q23.2	131798940	A	-0.132 (0.063)	3.73E-02	-0.167	2.85E-08	0.538	0
rs9483280	*ARG1*	6q23.2	131798329	G	-0.127 (0.063)	4.40E-02	-0.166	3.08E-08	0.486	0
rs9402328	*ARG1*	6q23.2	131798744	C	-0.127 (0.063)	4.40E-02	-0.166	3.08E-08	0.486	0
rs9492976	*ARG1*	6q23.2	131797691	A	-0.127 (0.063)	4.40E-02	-0.166	3.13E-08	0.486	0
rs2143779	*ARG1*	6q23.2	131800478	T	-0.125 (0.063)	4.73E-02	-0.165	3.41E-08	0.467	0
rs2179771	*ARG1*	6q23.2	131796623	C	-0.124 (0.063)	4.92E-02	-0.165	3.72E-08	0.465	0
rs9483281	*ARG1*	6q23.2	131806872	A	-0.117 (0.063)	6.35E-02	-0.163	5.35E-08	0.406	0
rs2608951	*ARG1*	6q23.2	131825473	G	-0.110 (0.062)	7.74E-02	-0.159	5.79E-08	0.361	0
rs2608928	*ARG1*	6q23.2	131833564	A	-0.114 (0.062)	6.76E-02	-0.160	6.27E-08	0.406	0
rs2608935	*ARG1*	6q23.2	131831429	A	-0.122 (0.063)	5.32E-02	-0.160	6.56E-08	0.490	0
rs9321294	*ARG1*	6q23.2	131837250	T	-0.114 (0.062)	6.76E-02	-0.159	6.70E-08	0.410	0
rs2491204	*ARG1*	6q23.2	131839097	T	-0.114 (0.062)	6.95E-02	-0.159	6.73E-08	0.404	0
rs2608927	*ARG1*	6q23.2	131835736	T	-0.114 (0.062)	6.76E-02	-0.159	6.85E-08	0.411	0
rs6904726	*ARG1*	6q23.2	131810390	A	-0.111 (0.063)	7.81E-02	-0.160	9.44E-08	0.380	0
rs6904733	*ARG1*	6q23.2	131810402	T	-0.111 (0.063)	7.87E-02	-0.160	9.54E-08	0.379	0
rs6927792	*ARG1*	6q23.2	131810800	C	-0.111 (0.063)	7.87E-02	-0.160	9.54E-08	0.379	0
rs2807272	*ARG1*	6q23.2	131832938	T	-0.117 (0.064)	6.95E-02	-0.148	1.30E-06	0.576	0
rs2807273	*ARG1*	6q23.2	131834214	G	-0.117 (0.064)	6.95E-02	-0.147	1.45E-06	0.584	0
rs4443525	*ARG1*	6q23.2	131839960	A	-0.116 (0.064)	7.14E-02	-0.146	1.79E-06	0.591	0
rs2608940	*ARG1*	6q23.2	131840624	T	-0.116 (0.064)	7.14E-02	-0.146	1.79E-06	0.591	0
rs2491209	*ARG1*	6q23.2	131822823	A	-0.093 (0.066)	1.58E-01	-0.144	4.01E-06	0.387	0
rs3861462	*ARG1*	6q23.2	131851129	C	-0.034 (0.058)	5.57E-01	-0.124	4.06E-06	0.083	66.84
rs9493000	*ARG1*	6q23.2	131850284	C	-0.027 (0.058)	6.43E-01	-0.123	4.56E-06	0.060	71.66
rs7740929	*ARG1*	6q23.2	131850315	G	-0.027 (0.058)	6.43E-01	-0.123	4.56E-06	0.060	71.66
rs9492992	*ARG1*	6q23.2	131844120	T	-0.002 (0.057)	9.74E-01	-0.118	1.05E-05	0.022	81.07
rs2608913	*ARG1*	6q23.2	131911954	C	-0.019 (0.043)	6.64E-01	-0.089	2.00E-05	0.063	70.98

rs2446213	*ARG1*	6q23.2	131915724	C	-0.012 (0.043)	7.75E-01	-0.084	4.75E-05	0.057	72.4
rs2608917	*ARG1*	6q23.2	131913649	G	-0.011 (0.043)	8.04E-01	-0.084	4.76E-05	0.052	73.55
rs2608919	*ARG1*	6q23.2	131915028	T	-0.012 (0.043)	7.75E-01	-0.084	4.91E-05	0.058	72.28
rs2608921	*ARG1*	6q23.2	131915428	T	-0.012 (0.043)	7.75E-01	-0.084	4.91E-05	0.058	72.28
rs2608915	*ARG1*	6q23.2	131913007	G	-0.012 (0.043)	7.80E-01	-0.084	4.96E-05	0.057	72.39
rs2608916	*ARG1*	6q23.2	131913298	G	-0.012 (0.043)	7.80E-01	-0.084	4.96E-05	0.057	72.39
rs2608918	*ARG1*	6q23.2	131914585	T	-0.012 (0.043)	7.80E-01	-0.084	4.96E-05	0.057	72.39
rs2608912	*ARG1*	6q23.2	131911476	C	-0.012 (0.043)	7.84E-01	-0.084	5.10E-05	0.057	72.5
rs2608914	*ARG1*	6q23.2	131912304	A	-0.012 (0.043)	7.84E-01	-0.084	5.10E-05	0.057	72.5
rs2608982	*ARG1*	6q23.2	131917893	T	-0.012 (0.043)	7.75E-01	-0.084	5.23E-05	0.059	72.04
rs2781650	*ARG1*	6q23.2	131918414	G	-0.012 (0.043)	7.75E-01	-0.084	5.23E-05	0.059	72.04
rs2781651	*ARG1*	6q23.2	131918636	G	-0.012 (0.043)	7.75E-01	-0.084	5.23E-05	0.059	72.04
rs2749933	*ARG1*	6q23.2	131918926	T	-0.012 (0.043)	7.75E-01	-0.084	5.23E-05	0.059	72.04
rs2608976	*ARG1*	6q23.2	131919868	C	-0.012 (0.043)	7.75E-01	-0.084	5.23E-05	0.059	72.04
rs2062375	*TNFRSF11B*	8q24.12	120046973	G	0.012 (0.045)	7.87E-01	0.089	3.77E-05	0.049	74.25
rs9969672	*TNFRSF11B*	8q24.12	120075261	T	0.014 (0.045)	7.56E-01	0.090	4.17E-05	0.053	73.25
rs11992136	*TNFRSF11B*	8q24.12	120072908	C	0.015 (0.045)	7.44E-01	0.089	4.35E-05	0.056	72.61
rs7014574	*TNFRSF11B*	8q24.12	120046258	C	0.007 (0.044)	8.74E-01	0.087	6.06E-05	0.040	76.29
rs2062377	*TNFRSF11B*	8q24.12	120076601	T	0.004 (0.045)	9.34E-01	0.085	9.39E-05	0.038	76.9
rs796127	*ARHGAP12*	10p11.22	32008735	T	-0.041 (0.042)	3.22E-01	-0.093	3.23E-06	0.158	49.89
rs796126	*ARHGAP12*	10p11.22	32008765	T	-0.040 (0.042)	3.43E-01	-0.093	3.26E-06	0.146	52.73
rs1775437	*ARHGAP12*	10p11.22	32004232	T	-0.038 (0.042)	3.59E-01	-0.092	4.50E-06	0.143	53.44
rs2488384	*ARHGAP12*	10p11.22	32013773	T	-0.035 (0.043)	4.15E-01	-0.093	5.31E-06	0.123	57.97
rs796130	*ARHGAP12*	10p11.22	32006513	G	-0.038 (0.042)	3.64E-01	-0.091	5.64E-06	0.149	51.94
rs796082	*ZEB1*	10p11.22	31960297	G	-0.018 (0.043)	6.72E-01	-0.083	3.91E-05	0.085	66.36
rs2393791	*HNF1A*	12q24.31	119908339	C	-0.138 (0.041)	8.28E-04	-0.122	2.90E-10	0.665	0

rs7310409	*HNF1A*	12q24.31	119909244	A	-0.138 (0.041)	7.79E-04	-0.121	4.35E-10	0.641	0
rs2393775	*HNF1A*	12q24.31	119908957	G	-0.137 (0.042)	1.08E-03	-0.120	7.51E-10	0.647	0
rs1169313	*C12orf43*	12q24.31	119927053	T	0.109 (0.041)	7.96E-03	0.117	1.72E-09	0.828	0
rs1169310	*HNF1A*	12q24.31	119923816	G	0.104 (0.041)	1.08E-02	0.117	1.75E-09	0.716	0
rs1169306	*HNF1A*	12q24.31	119922694	C	0.104 (0.041)	1.08E-02	0.117	1.84E-09	0.719	0
rs735396	*HNF1A*	12q24.31	119923227	T	0.104 (0.041)	1.08E-02	0.117	1.91E-09	0.721	0
rs2464195	*HNF1A*	12q24.31	119919858	G	0.104 (0.041)	1.08E-02	0.117	2.09E-09	0.726	0
rs2259816	*HNF1A*	12q24.31	119919970	G	0.104 (0.041)	1.04E-02	0.116	2.45E-09	0.751	0
rs2259820	*HNF1A*	12q24.31	119919725	C	0.097 (0.041)	1.79E-02	0.114	5.13E-09	0.630	0
rs2464196	*HNF1A*	12q24.31	119919810	G	0.097 (0.041)	1.79E-02	0.114	5.13E-09	0.630	0
rs1182933	*C12orf43*	12q24.31	119939005	C	0.093 (0.042)	2.72E-02	0.109	3.44E-08	0.656	0
rs1920792	*HNF1A*	12q24.31	119888967	C	0.118 (0.040)	3.56E-03	0.103	8.12E-08	0.680	0
rs7305618	*HNF1A*	12q24.31	119887315	T	0.118 (0.040)	3.56E-03	0.103	8.79E-08	0.675	0
rs2708101	*OASL*	12q24.31	119972015	C	0.091 (0.041)	2.51E-02	0.103	1.03E-07	0.750	0
rs7953249	*HNF1A*	12q24.31	119888107	A	0.118 (0.040)	3.53E-03	0.102	1.27E-07	0.645	0
rs1169303	*HNF1A*	12q24.31	119920759	A	0.095 (0.046)	3.79E-02	0.110	1.04E-06	0.726	0
rs17596685	*TNFSF11*	13q14.11	42015201	T	-0.059 (0.043)	1.74E-01	-0.093	3.74E-06	0.378	0

*Among the 98 SNP loci with unadjusted *P* value < 10^{-5} in stage 1 result, 92 available SNPs in stage 2 data were presented

Table S4. *P*-values and LD values of neighboring SNPs of the rs9375813 in the *ARG1* locus.

L1	L2	Gene	BP	BETA	SE	P-value*	D'	r^2
rs9375813	rs17596690	*ARG1*	131794917	-0.152	0.035	1.42E-05	0.963	0.874
rs9375813	rs2179771	*ARG1*	131796623	-0.177	0.034	2.14E-07	1	0.999
rs9375813	rs9492976	*ARG1*	131797691	-0.177	0.034	2.06E-07	1	0.999
rs9375813	rs9483280	*ARG1*	131798329	-0.177	0.034	2.02E-07	1	0.999
rs9375813	rs9402328	*ARG1*	131798744	-0.177	0.034	2.02E-07	1	0.999
rs9375813	rs2143779	*ARG1*	131800478	-0.177	0.034	2.05E-07	1	0.999
rs9375813	rs2608903	*ARG1*	131805071	-0.076	0.024	1.35E-03	0.998	0.260
rs9375813	rs9483281	*ARG1*	131806872	-0.176	0.034	2.31E-07	0.999	0.999
rs9375813	rs6904726	*ARG1*	131810390	-0.174	0.034	3.26E-07	0.999	0.999
rs9375813	rs6904733	*ARG1*	131810402	-0.174	0.034	3.26E-07	0.999	0.999
rs9375813	rs6927792	*ARG1*	131810800	-0.174	0.034	3.26E-07	0.999	0.999
rs9375813	rs2491209	*ARG1*	131822823	-0.158	0.035	7.82E-06	0.988	0.923
rs9375813	rs2608951	*ARG1*	131825473	-0.174	0.033	1.96E-07	0.996	0.951
rs9375813	rs2608935	*ARG1*	131831429	-0.171	0.034	3.68E-07	0.995	0.940
rs9375813	rs2807272	*ARG1*	131832938	-0.157	0.035	6.30E-06	0.969	0.903
rs9375813	rs2608928	*ARG1*	131833564	-0.173	0.034	2.54E-07	0.997	0.960
rs9375813	rs2807273	*ARG1*	131834214	-0.156	0.035	7.10E-06	0.968	0.903
rs9375813	rs2608927	*ARG1*	131835736	-0.172	0.034	2.80E-07	0.997	0.959
rs9375813	rs9321294	*ARG1*	131837250	-0.173	0.034	2.75E-07	0.997	0.959
rs9375813	rs2491204	*ARG1*	131839097	-0.173	0.034	2.69E-07	0.997	0.958
rs9375813	rs4443525	*ARG1*	131839960	-0.155	0.035	8.64E-06	0.968	0.902
rs9375813	rs2608940	*ARG1*	131840624	-0.155	0.035	8.64E-06	0.968	0.902

Table S5. Associations of the previously reported CRP-related loci.

SNP	Chr	Position* (bp)	Nearest Gene	MAF	Type**	Minor allele	Effect Size	*P*	Reference
rs6700896	1	65862370	*LEPR*	0.11	imputed	C	-0.017	0.6171	Elliot et al, 2009
rs1805096	1	65874845	*LEPR*	0.11	genotyped	G	-0.017	0.6164	Reiner et al, 2012
rs1892534	1	65878532	*LEPR*	0.11	genotyped	C	-0.018	0.6002	Ridker et al, 2008
rs2186245	1	65890652	*LEPR*	0.24	imputed	C	-0.018	0.4948	Ridker et al, 2008
rs12022410	1	65926521	*LEPR*	0.09	imputed	A	-0.051	0.1911	Ridker et al, 2008
rs2211651	1	65928609	*LEPR*	0.11	imputed	G	-0.015	0.6609	Ridker et al, 2008
rs2889195	1	65929318	*LEPR*	0.11	genotyped	C	-0.012	0.7178	Ridker et al, 2008
rs4291477	1	65963663	*PDE4B*	0.17	genotyped	G	-0.029	0.3151	Ridker et al, 2008
rs4537545	1	152685503	*IL6R*	0.44	genotyped	T	-0.056	0.0112	Elliot et al, 2009
rs4129267	1	152692888	*IL6R*	0.44	imputed	T	-0.059	0.0089	Ridker et al, 2008
rs8192284	1	152693594	*IL6R*	0.44	imputed	C	-0.059	0.0087	Ridker et al, 2008; Okada et al, 2011
rs12093699	1	157914612	*CRP*	0.06	imputed	A	0.147	0.0017	Ridker et al, 2008
rs876537	1	157941557	*CRP*	0.36	imputed	C	0.182	3.61E-15	Wu et al, 2012
rs2794520	1	157945440	*CRP*	0.36	imputed	C	0.181	2.82E-15	Ridker et al, 2008
rs3093075	1	157946537	*CRP*	0.15	imputed	T	0.190	8.66E-10	Ridker et al, 2008
rs1205	1	157948857	*CRP*	0.36	imputed	C	0.181	2.21E-15	Ridker et al, 2008
rs1417938	1	157950810	*CRP*	0.07	imputed	A	0.169	9.89E-05	Ridker et al, 2008

rs2027471	1	157956012	*CRP*	0.34	imputed	T	0.171	2.30E-13	Ridker et al, 2008
rs3116656	1	157958996	*CRP*	0.07	imputed	G	0.182	1.58E-05	Ridker et al, 2008
rs12068753	1	157959161	*CRP*	0.14	genotyped	A	0.197	3.41E-10	Ridker et al, 2008
rs3116653	1	157963534	*CRP*	0.08	imputed	C	0.173	2.98E-05	Ridker et al, 2008
rs3116651	1	157965109	*CRP*	0.08	genotyped	T	0.171	3.44E-05	Ridker et al, 2008
rs7553007	1	157965173	*CRP*	0.37	genotyped	G	0.182	7.34E-16	Ridker et al, 2008; Elliot et al, 2009; Reiner et al, 2012
rs780094	2	27594741	*GCKR*	0.46	genotyped	C	-0.059	0.0067	Ridker et al, 2008
rs1260333	2	27602128	*GCKR*	0.46	imputed	G	-0.062	0.0054	Ridker et al, 2008
rs2097677	7	22699364	*IL6*	0.24	imputed	A	0.091	0.0004	Okada et al, 2011
rs10778213	12	102019281	*ASCL1*	0.17	imputed	T	0.055	0.0607	Ridker et al, 2008
rs7305618	12	119887315	*HNF1A*	0.47	imputed	T	0.099	6.86E-06	Wu et al, 2012
rs7953249	12	119888107	*HNF1A*	0.47	genotyped	A	0.097	9.85E-06	Ridker et al, 2008
rs7310409	12	119909244	*HNF1A*	0.48	imputed	A	-0.116	1.38E-07	Ridker et al, 2008; Okada et al, 2011
rs2464196	12	119919810	*HNF1A*	0.45	imputed	G	0.119	8.58E-08	Ridker et al, 2008
rs2259816	12	119919970	*HNF1A*	0.45	genotyped	G	0.119	7.10E-08	Reiner et al, 2012
rs1169307	12	119922765	*HNF1A*	0.10	imputed	T	0.131	0.0003	Ridker et al, 2008
rs735396	12	119923227	*HNF1A*	0.44	imputed	T	0.121	5.34E-08	Ridker et al, 2008
rs4420638	19	50114786	*APOE-CI-CII cluster*	0.11	genotyped	G	-0.098	0.0040	Elliot et al, 2009; Okada et al, 2011

*Position is based on NCBI human genome build 36.3 (hg18)

**Type indicates if a SNP is genotype or imputed; Chr, Chromosome; MAF, minor allele frequency

Table S6. Re-prioritized genetic variants list after GWAS.

SNPs	Chr	Position	GWAS P-value	Final Weight	Before Re-prioritization	After Re-prioritization
rs7553007	1	157965173	7.34E-16	5.40E-16	1	1
rs1205	1	157948857	2.21E-15	2.27E-15	2	2
rs2794520	1	157945440	2.82E-15	2.49E-15	4	3
rs2808629	1	157943420	2.50E-15	3.27E-15	3	4
rs2808628	1	157942635	3.43E-15	4.48E-15	5	5
rs876537	1	157941557	3.61E-15	4.72E-15	6	6
rs2027471	1	157956012	2.30E-13	3.01E-13	7	7
rs1341665	1	157958183	2.98E-13	3.89E-13	8	8
rs16842599	1	157964099	2.39E-10	2.46E-10	9	9
rs12081480	1	157973614	2.96E-10	3.05E-10	12	10
rs16842568	1	157942844	2.54E-10	3.32E-10	10	11
rs16842559	1	157942795	2.54E-10	3.32E-10	11	12
rs12081252	1	157973137	3.28E-10	3.38E-10	13	13
rs12081264	1	157973184	3.81E-10	3.92E-10	15	14
rs12068753	1	157959161	3.41E-10	4.46E-10	14	15
**rs2608912**	**6**	**131911476**	**7.94E-06**	**7.22E-10**	**87**	**16**
**rs2608976**	**6**	**131919868**	**8.52E-06**	**7.62E-10**	**88**	**17**
rs3093075	1	157946537	8.66E-10	1.13E-09	16	18
**rs2608921**	**6**	**131915428**	**7.89E-06**	**1.21E-09**	**81**	**19**
rs3093077	1	157946260	9.30E-10	1.22E-09	17	20
rs16842502	1	157920487	7.80E-09	1.02E-08	18	21
rs1169310	12	119923816	4.95E-08	5.10E-08	19	22
rs1169306	12	119922694	5.15E-08	5.30E-08	20	23
rs735396	12	119923227	5.34E-08	5.50E-08	21	24
rs2464195	12	119919858	5.83E-08	6.00E-08	22	25
rs1169313	12	119927053	6.66E-08	6.86E-08	23	26
rs2259816	12	119919970	7.10E-08	7.31E-08	24	27
rs2464196	12	119919810	8.58E-08	8.83E-08	25	28
rs2259820	12	119919725	8.58E-08	8.83E-08	26	29
rs2393791	12	119908339	8.89E-08	9.15E-08	27	30
rs7310409	12	119909244	1.38E-07	1.42E-07	28	31
rs2393775	12	119908957	1.75E-07	1.80E-07	29	32
rs1891187	1	157895677	2.34E-07	2.07E-07	42	33
rs4275453	1	157974671	2.06E-07	2.12E-07	36	34
rs1446976	1	157895818	2.32E-07	2.13E-07	41	35
rs1891186	1	157895645	2.34E-07	2.32E-07	43	36
rs2794526	1	157892102	1.79E-07	2.34E-07	30	37
rs2608951	6	131825473	1.96E-07	2.56E-07	31	38
rs9402328	6	131798744	2.02E-07	2.64E-07	32	39
rs9483280	6	131798329	2.02E-07	2.64E-07	33	40
rs2143779	6	131800478	2.05E-07	2.68E-07	34	41
rs9492976	6	131797691	2.06E-07	2.69E-07	35	42
rs2179771	6	131796623	2.14E-07	2.80E-07	37	43
rs9375813	6	131798940	2.30E-07	3.01E-07	38	44
rs9483281	6	131806872	2.31E-07	3.02E-07	39	45
rs1446975	1	157895966	2.32E-07	3.03E-07	40	46

rs2608928	6	131833564	2.54E-07	3.32E-07	44	47
rs2491204	6	131839097	2.69E-07	3.52E-07	45	48
rs9321294	6	131837250	2.75E-07	3.59E-07	46	49
rs2608927	6	131835736	2.80E-07	3.66E-07	47	50
rs1182933	12	119939005	4.05E-07	4.17E-07	52	51
rs6927792	6	131810800	3.26E-07	4.26E-07	48	52
rs6904733	6	131810402	3.26E-07	4.26E-07	49	53
rs6904726	6	131810390	3.26E-07	4.26E-07	50	54
rs2608935	6	131831429	3.68E-07	4.81E-07	51	55
rs12087465	1	157891923	4.17E-07	5.45E-07	53	56
rs9492992	6	131844120	6.88E-07	8.99E-07	54	57
rs2794500	1	157901645	7.94E-07	1.04E-06	55	58
rs7740929	6	131850315	8.41E-07	1.10E-06	56	59
rs9493000	6	131850284	8.41E-07	1.10E-06	57	60
rs3861462	6	131851129	1.04E-06	1.36E-06	58	61
rs2708101	12	119972015	1.36E-06	1.40E-06	59	62
rs796126	10	32008765	1.76E-06	2.30E-06	60	63
rs796127	10	32008735	1.95E-06	2.55E-06	61	64
rs2488384	10	32013773	2.20E-06	2.88E-06	62	65
rs1775437	10	32004232	2.35E-06	3.07E-06	63	66
rs2608913	6	131911954	3.76E-06	3.87E-06	65	67
rs796130	10	32006513	2.97E-06	3.88E-06	64	68
rs1841972	5	121824037	4.87E-06	6.36E-06	66	69
rs1920792	12	119888967	6.35E-06	6.54E-06	70	70
rs2062375	8	120046973	5.22E-06	6.82E-06	67	71
rs7305618	12	119887315	6.86E-06	7.06E-06	74	72
rs2608917	6	131913649	6.93E-06	7.14E-06	76	73
rs17596685	13	42015201	6.69E-06	7.47E-06	71	74
rs2446213	6	131915724	7.55E-06	7.77E-06	79	75
rs2608919	6	131915028	7.89E-06	8.12E-06	82	76
rs2608918	6	131914585	7.93E-06	8.17E-06	83	77
rs2608916	6	131913298	7.93E-06	8.17E-06	84	78
rs2608915	6	131913007	7.93E-06	8.17E-06	85	79
rs2608914	6	131912304	7.94E-06	8.18E-06	86	80
rs9969672	8	120075261	6.29E-06	8.22E-06	68	81
rs2807272	6	131832938	6.30E-06	8.23E-06	69	82
rs2749933	6	131918926	8.52E-06	8.77E-06	89	83
rs2781651	6	131918636	8.52E-06	8.77E-06	90	84
rs2781650	6	131918414	8.52E-06	8.77E-06	91	85
rs2608982	6	131917893	8.52E-06	8.77E-06	92	86
rs7014574	8	120046258	6.78E-06	8.86E-06	72	87
rs10478555	5	121809430	6.78E-06	8.86E-06	73	88
rs11992136	8	120072908	6.92E-06	9.04E-06	75	89
rs12517578	5	106008730	6.98E-06	9.12E-06	77	90
rs2807273	6	131834214	7.10E-06	9.28E-06	78	91
rs1169303	12	119920759	9.47E-06	9.75E-06	96	92
rs7953249	12	119888107	9.85E-06	1.01E-05	98	93
rs2491209	6	131822823	7.82E-06	1.02E-05	80	94
rs2608940	6	131840624	8.64E-06	1.13E-05	93	95
rs4443525	6	131839960	8.64E-06	1.13E-05	94	96

rs796082	10	31960297	9.24E-06	1.21E-05	95	97
rs2062377	8	120076601	9.85E-06	1.29E-05	97	98
rs1978838	5	106008311	1.01E-05	1.32E-05	99	99
rs7010267	8	120015837	1.05E-05	1.37E-05	100	100

Bold characters represent three SNPs, rs2608912, rs2608976, rs2608921, whose ranking of significance levels became highly elevated after SNP prioritization from 87th, 88th and 81th to 16th, 17th and 19th, respectively.

Table S7. Associations of the top significant SNP polymorphisms on cardiovascular disease traits based on the whole KARE samples (7,626 samples).

Disease Trait	N[¶]	Chr	SNP	Position	Minor Allele	*P*[*]	OR[†]	SE	L95[‡]	U95[§]
Myocardial infarction	55	1	rs7553007	157965173	G	0.1566	1.31	0.19	0.90	1.92
		6	rs9375813	131798940	A	0.3262	0.71	0.35	0.36	1.41
		12	rs2393791	119908339	C	0.3223	1.21	0.19	0.83	1.77
Coronary artery disease	65	1	rs7553007	157965173	G	0.9496	1.01	0.18	0.71	1.45
		6	rs9375813	131798940	A	0.3253	0.73	0.32	0.39	1.36
		12	rs2393791	119908339	C	0.1322	0.76	0.18	0.54	1.09
Hypertension	1115	1	rs7553007	157965173	G	0.6138	1.03	0.05	0.93	1.13
		**6**	**rs9375813**	**131798940**	**A**	**0.0440**	**0.86**	**0.08**	**0.74**	**1.00**
		12	rs2393791	119908339	C	0.5570	0.97	0.05	0.89	1.07

[¶]N : Number of case samples

[*]*P*: Age and gender adjusted logistic regression was performed based on the above phenotype.

[†]OR: Estimated odds ratio for minor allele

[‡]L95: Lower bound of 95% confidence interval for odds ratio

[§]U95: Upper bound of 95% confidence interval for odds ratio

Table S8. Genes mapped with variant in adaptive immune response.

Gene Name	Variant	Functional Class*	$-\log_{10}$(*P*-value)**
CRP	rs7553007	-	15.134
EXO1	rs1418761	-	3.079
TLR4	rs2244872	-	2.409
C9	rs2738238	-	2.407
CTSC	rs217090	-	2.363
SYK	rs2613310	-	2.281
CD46	rs7144	-	2.121
PRKCD	rs13084863	-	2
IL12B	rs6896881	-	1.965
TXK	rs10938521	-	1.85
C8B	rs599857	-	1.803
CR1	rs677066	-	1.797
MSH2	rs6708696	-	1.744
PIK3CD	rs4240896	-	1.734
RIPK2	rs2665636	-	1.726
PIK3CG	rs7804699	-	1.718
CADM1	rs693246	-	1.692
C5	rs2159777	-	1.648
IFNG	rs4244065	-	1.614
RAB27A	rs9920114	-	1.608
IFNB1	rs4977657	-	1.551
JAM3	rs661820	-	1.535
LIG4	rs16971751	-	1.53
ITK	rs25856	-	1.527
MEF2C	rs4521516	-	1.496
KLHL6	rs7618957	-	1.418
C7	rs2455307	-	1.411
CR2	rs7527218	-	1.408
HSPD1	rs13386066	-	1.407
MBL2	rs12262251	-	1.401
VEGFA	rs4714696	-	1.386
RORC	rs3828057	-	1.368
POU2F2	rs11883412	-	1.355
GCNT3	rs6494120	-	1.351

LYST	rs12038267	-	1.332
SERPING1	rs2729358	-	1.326
ZAP70	rs2290127	-	1.323
CLU	rs1316801	-	1.305
C3	rs11569538	-	1.303
MLH1	rs1540354	-	1.232
IL18	rs360729	-	1.207
EXOSC3	rs10814621	-	1.156
CTSH	rs12438191	-	1.126
FAS	rs17378702	-	1.107
BCL6	rs873205	-	1.04
TNFSF4	rs16845607	-	1.03
CXCL13	rs1500498	-	0.973
IL4	rs2243283	-	0.962
CTSS	rs12568757	-	0.922
C8A	rs635833	-	0.888
C6	rs3805716	-	0.874
NBN	rs1805793	-	0.858
LEF1	rs11097978	-	0.827
NOD2	rs1981760	-	0.819
DLG1	rs768858	-	0.811
C4BPA	rs10746451	-	0.758
TNFRSF11A	rs3744929	-	0.725
CD55	rs2940252	-	0.723
C2	rs9267673	-	0.717
C1RL	rs744141	-	0.691
AICDA	rs4883169	-	0.683
XRCC4	rs6894425	-	0.677
HLA-DRB1	rs615672	-	0.63
CFI	rs4698788	-	0.621
MASP2	rs7556039	-	0.618
TGFB1	rs2241716	-	0.616
FCER1G	rs4489574	-	0.609
SWAP70	rs10770049	-	0.593
UNC13D	rs8067076	-	0.592
C1S	rs7306673	-	0.568
CD74	rs2569094	-	0.565

LY9	rs4656932	-	0.539
BCL3	rs8100239	-	0.529
INPP5D	rs3935584	-	0.526
BCL10	rs11161585	-	0.502
CRTAM	rs7126620	-	0.419
MSH6	rs17036815	-	0.398
HLA-DQB1	rs35120848	-	0.344
C1QA	rs158762	-	0.337
TLR6	rs3775073	-	0.334
CD8A	rs1515950	-	0.307
C3AR1	rs10846450	-	0.28
SLC11A1	rs13062	-	0.267
ADAM17	rs10929587	-	0.264
MYD88	rs7744	-	0.232
SLA2	rs221308	-	0.224
HLA-DMA	rs1050391	-	0.221
ZP3	rs1019096	-	0.207
NFKB2	rs11574851	-	0.194
C1QB	rs629409	-	0.181
C4BPB	rs8942	-	0.137
STAT6	rs12368672	-	0.129
CTSL1	rs2274611	-	0.059

[*]Functional class, SNPs that may alter protein, gene expression or the role of protein in context of pathway

[**]-log10(P) for SNP in discovery phase of this GWAS

Table S9. Genes mapped with variant in leukocyte mediated immunity.

Gene Name	Variant	Functional Class	-log10(P-value)
CRP	rs7553007	-	15.134
IL6	rs2961312	-	3.197
EXO1	rs1418761	-	3.079
TLR4	rs2244872	-	2.409
C9	rs2738238	-	2.407
CTSC	rs217090	-	2.363
CLEC2A	rs7957464	-	2.361
CPLX2	rs2443541	-	2.313
CD46	rs7144	-	2.121
PRKCD	rs13084863	-	2
IL6R	rs4537545	-	1.952
C8B	rs599857	-	1.803
CR1	rs677066	-	1.797
MSH2	rs6708696	-	1.744
PIK3CD	rs4240896	-	1.734
PIK3CG	rs7804699	-	1.718
CADM1	rs693246	-	1.692
YWHAZ	rs1470764	-	1.688
C5	rs2159777	-	1.648
RAB27A	rs9920114	-	1.608
LIG4	rs16971751	-	1.53
C7	rs2455307	-	1.411
MYO1F	rs12462590	-	1.41
CR2	rs7527218	-	1.408
HSPD1	rs13386066	-	1.407
MBL2	rs12262251	-	1.401
ANXA3	rs6855458	-	1.376
POU2F2	rs11883412	-	1.355
GCNT3	rs6494120	-	1.351
LYST	rs12038267	-	1.332
SERPING1	rs2729358	-	1.326
CLU	rs1316801	-	1.305
C3	rs11569538	-	1.303

MLL5	rs10274607	-	1.254
MLH1	rs1540354	-	1.232
EXOSC3	rs10814621	-	1.156
DBH	rs3025425	-	1.137
CTSH	rs12438191	-	1.126
FAS	rs17378702	-	1.107
TUBB	rs8233	-	1.094
SERPINB9	rs318486	-	1.077
TNFSF4	rs16845607	-	1.03
CTSG	rs17257069	-	0.994
IL4	rs2243283	-	0.962
C8A	rs635833	-	0.888
ADORA3	rs2798564	-	0.874
C6	rs3805716	-	0.874
NBN	rs1805793	-	0.858
NOD2	rs1981760	-	0.819
DLG1	rs768858	-	0.811
STXBP3	rs11102337	-	0.773
C4BPA	rs10746451	-	0.758
CD55	rs2940252	-	0.723
C2	rs9267673	-	0.717
PLA2G1B	rs2522136	-	0.709
C1RL	rs744141	-	0.691
AICDA	rs4883169	-	0.683
XRCC4	rs6894425	-	0.677
SLAMF7	rs17378627	-	0.667
HLA-DRB1	rs615672	-	0.63
CFI	rs4698788	-	0.621
MASP2	rs7556039	-	0.618
FCER1G	rs4489574	-	0.609
SWAP70	rs10770049	-	0.593
UNC13D	rs8067076	-	0.592
C1S	rs7306673	-	0.568
CD74	rs2569094	-	0.565
LY9	rs4656932	-	0.539
BCL3	rs8100239	-	0.529
INPP5D	rs3935584	-	0.526

BCL10	rs11161585	-	0.502
CRTAM	rs7126620	-	0.419
MSH6	rs17036815	-	0.398
LYN	rs16922296	-	0.385
ACE	rs4351	-	0.358
HLA-DQB1	rs35120848	-	0.344
C1QA	rs158762	-	0.337
CD8A	rs1515950	-	0.307
LAT	rs9924619	-	0.305
CEBPG	rs17530508	-	0.299
SLC11A1	rs13062	-	0.267
ADAM17	rs10929587	-	0.264
MYD88	rs7744	-	0.232
SLA2	rs221308	-	0.224
HLA-DMA	rs1050391	-	0.221
ZP3	rs1019096	-	0.207
C1QB	rs629409	-	0.181
C4BPB	rs8942	-	0.137

Table S10. Genes mapped with variant in photoreceptor outer segment.

Gene Name	Variant	Functional Class	$-\log_{10}$(*P*-value)
HNF1A	rs2259816	-	7.149
RAPGEF4	rs1530591	-	2.877
MAK	rs478791	-	2.799
RP1	rs9650182	-	2.289
MYRIP	rs17759069	-	2.145
PROM1	rs12152560	-	2.032
GNAT3	rs10954212	-	1.987
PCDH15	rs11003592	-	1.972
RP1L1	rs4427133	-	1.627
RAB27A	rs9920114	-	1.608
PTPRK	rs41514350	-	1.501
CNGA1	rs7694104	-	1.454
GNB1	rs2272908	-	1.401
TULP1	rs3777746	-	1.341
CNGB3	rs11988618	-	1.229
MYO5A	rs1693523	-	1.214
RHO	rs2855552	-	0.958
PDC	rs11803944	-	0.804
CNGA3	rs4851131	-	0.791
CDHR1	rs10509491	-	0.765
INHA	rs753974	-	0.558
PTGS1	rs1234909	-	0.454
MYO7A	rs3781695	-	0.452
C2orf71	rs17744052	-	0.432
MERTK	rs13016143	-	0.348
IQCB1	rs4311252	-	0.184
PPEF2	rs13434876	-	0.122

Table S11. Genes mapped with variant in cell surface binding.

Gene Name	Variant	Functional class	$-\log_{10}$(*P*-value)
CRP	rs7553007	-	15.134
PCSK6	rs1947942	-	2.162
CD36	rs17260734	-	2.045
PRB3	rs1834856	-	2.022
THBS1	rs2618162	-	1.644
SFTPD	rs2181204	-	1.551
STAB2	rs1866297	-	1.527
AIMP1	rs16999761	-	1.524
SELP	rs3917843	-	1.465
SELPLG	rs11114011	-	1.441
HSPD1	rs13386066	-	1.407
MBL2	rs12262251	-	1.401
VEGFA	rs4714696	-	1.386
PDGFC	rs2343116	-	1.376
BPI	rs7273966	-	1.322
ANXA5	rs4833753	-	1.32
SPN	rs1364182	-	1.3
F3	rs10782985	-	1.287
APOH	rs2873966	-	1.269
AGGF1	rs3762982	-	1.194
APOA4	rs1263173	-	1.038
TLR2	rs11938228	-	0.908
ATP5A1	rs9955008	-	0.881
LBP	rs2232596	-	0.753
PLA2G1B	rs2522136	-	0.709
HBEGF	rs2237077	-	0.631
CD1D	rs400214	-	0.631
CD48	rs352683	-	0.619
TGFB1	rs2241716	-	0.616
CD2	rs797999	-	0.603
ADIPOQ	rs6773957	-	0.518
DAG1	rs1050088	-	0.338

PLG	rs2144723	-	0.285
PGLYRP1	rs1861790	-	0.256
PDGFB	rs17000920	-	0.161
FGB	rs2059503	-	0.15
HRG	rs16861013	-	0.148
SPACA3	rs28906	-	0.141
ALB	rs16849267	-	0.13
LILRB2	rs4022330	-	0.121
SERPINF2	rs4790285	-	0.048

Table S12. Genes mapped with variant in cholesterol binding

Gene Name	Variant	Functional Class	$-\log_{10}$(*P*-value)
CRP	rs7553007	-	15.134
ABCA1	rs4260948	-	2.749
SOAT1	rs425858	-	2.07
PTCH1	rs7862599	-	1.801
ABCG1	rs2839482	-	1.776
PMP2	rs6995156	-	1.41
APOA2	rs5085	-	1.139
NR1H3	rs7118396	-	0.74
CYP11A1	rs7174179	-	0.612
CAV1	rs1049334	-	0.557
NPC2	rs2358785	-	0.482
APOC3	rs2071523	-	0.389
SOAT2	rs12809590	-	0.323
TSPO2	rs9369260	-	0.315
STARD3	rs11869286	-	0.273
CETP	rs1800775	-	0.194
STAR	rs16887217	-	0.109
IHH	rs10208728	-	0.031

Table S13. Genes mapped with variant in bacterial cell surface binding

Gene Name	Variant	Functional Class	P-value
CRP	rs7553007	-	15.134
CD36	rs17260734	-	2.045
PRB3	rs1834856	-	2.022
SFTPD	rs2181204	-	1.551
STAB2	rs1866297	-	1.527
SELPLG	rs11114011	-	1.441
MBL2	rs12262251	-	1.401
BPI	rs7273966	-	1.322
SPN	rs1364182	-	1.3
TLR2	rs11938228	-	0.908
LBP	rs2232596	-	0.753
DAG1	rs1050088	-	0.338
PGLYRP1	rs1861790	-	0.256
SPACA3	rs28906	-	0.141

Table 14. Pathway analysis results of GSA-SNP software

set name	gene count	set size	z-score	p-value	corrected p-value	FDR
low-density lipoprotein particle binding	12	13	10.65696	0	0	<0.001
lipoprotein particle binding	20	21	9.056942	0	0	<0.001
protein-lipid complex binding	20	21	9.056942	0	0	<0.001
opsonization	7	7	7.509131	3.00E-14	4.84E-11	<0.001
negative regulation of lipid storage	14	14	7.456615	4.42E-14	5.71E-11	<0.001
bacterial cell surface binding	16	17	7.360872	9.16E-14	9.86E-11	<0.001
quaternary ammonium group binding	15	15	7.349194	1.00E-13	9.23E-11	<0.001
negative regulation of macrophage derived foam cell differentiation	13	13	7.251112	2.06E-13	1.66E-10	<0.001
regulation of lipid storage	30	31	6.647502	1.49E-11	1.07E-08	<0.001
humoral immune response mediated by circulating immunoglobulin	31	165	6.461015	5.20E-11	3.36E-08	<0.001
response to lead ion	13	14	6.119311	4.70E-10	2.76E-07	<0.001
complement activation, classical pathway	26	160	6.087637	5.73E-10	3.08E-07	<0.001
regulation of macrophage derived foam cell differentiation	27	27	6.064354	6.62E-10	3.29E-07	<0.001
humoral immune response	72	220	5.744094	4.62E-09	2.13E-06	<0.001
cholesterol binding	20	23	5.468741	2.27E-08	9.76E-06	<0.001
alcohol binding	39	42	5.385632	3.61E-08	1.46E-05	<0.001
sterol binding	27	30	5.211166	9.38E-08	3.57E-05	<0.001
presynaptic membrane	43	47	5.137044	1.40E-07	5.01E-05	<0.001
complement activation	33	174	4.856848	5.96E-07	2.03E-04	<0.001

interferon-gamma-mediated signaling pathway	58	69	4.723106	1.16E-06	3.75E-04	<0.001
regulation of bone resorption	24	26	4.606352	2.05E-06	6.30E-04	<0.001
regulation of inclusion body assembly	5	5	4.585766	2.26E-06	6.64E-04	<0.001
neuron cell-cell adhesion	12	12	4.54256	2.78E-06	7.80E-04	<0.001
regulation of axonogenesis	89	97	4.521542	3.07E-06	8.26E-04	<0.001
cell adhesion molecule binding	50	52	4.519113	3.10E-06	8.02E-04	<0.001
bone resorption	18	18	4.490447	3.55E-06	8.83E-04	<0.001
cellular response to interferon-gamma	73	84	4.472573	3.86E-06	9.25E-04	<0.001
immunoglobulin mediated immune response	57	194	4.468101	3.95E-06	9.10E-04	<0.001
bone remodeling	29	31	4.441771	4.46E-06	9.94E-04	<0.001
adherens junction organization	41	42	4.421697	4.90E-06	0.001054	<0.001
lymphocyte mediated immunity	78	216	4.38258	5.86E-06	0.001222	<0.001
B cell mediated immunity	59	196	4.368199	6.26E-06	0.001264	<0.001
ion gated channel activity	21	21	4.362452	6.43E-06	0.001259	<0.001
sarcoplasm	46	53	4.361622	6.46E-06	0.001226	<0.001
neuron recognition	26	28	4.348114	6.87E-06	0.001267	<0.001
Ras guanyl-nucleotide exchange factor activity	88	93	4.341321	7.08E-06	0.001271	<0.001
oxidoreductase activity, acting on paired donors, with oxidation of a pair of donors resulting in the reduction of molecular oxygen to two molecules of water	6	7	4.339601	7.14E-06	0.001246	<0.001
sensory perception of pain	47	50	4.286067	9.09E-06	0.001546	<0.001
regulation of gene expression by genetic imprinting	10	14	4.285719	9.11E-06	0.001509	<0.001
renal absorption	10	12	4.276583	9.49E-06	0.001533	<0.001
regulation of bone remodeling	27	29	4.244055	1.10E-05	0.001729	<0.001

renal system process	61	65	4.172091	1.51E-05	0.002321	<0.001
homophilic cell adhesion	89	142	4.133757	1.78E-05	0.002681	<0.001
regulation of ion transmembrane transporter activity	87	92	4.129602	1.82E-05	0.002668	<0.001
Rac GTPase activator activity	13	14	4.070027	2.35E-05	0.003374	<0.001
positive regulation of developmental growth	37	40	4.052437	2.53E-05	0.003559	<0.001
cell surface binding	49	54	4.022512	2.88E-05	0.003957	<0.001
regulation of ion transmembrane transport	99	104	4.004745	3.10E-05	0.004178	<0.001
regulation of developmental growth	88	95	3.995403	3.23E-05	0.004257	<0.001
genetic imprinting	14	18	3.977398	3.48E-05	0.004501	<0.001
protein activation cascade	51	194	3.973328	3.54E-05	0.004489	<0.001
sarcoplasmic reticulum	43	49	3.962999	3.70E-05	0.004597	<0.001
positive regulation of vascular endothelial growth factor receptor signaling pathway	15	16	3.951533	3.88E-05	0.004732	<0.001
M band	12	12	3.931123	4.23E-05	0.005057	<0.001
regulation of transmembrane transporter activity	91	97	3.913258	4.55E-05	0.005348	<0.001
activation of transmembrane receptor protein tyrosine kinase activity	9	10	3.902895	4.75E-05	0.005482	<0.001
regulation of calcium-mediated signaling	28	31	3.890484	5.00E-05	0.005669	<0.001
reverse cholesterol transport	15	17	3.887109	5.07E-05	0.005649	<0.001
positive regulation of calcium-mediated signaling	21	23	3.851229	5.88E-05	0.006434	<0.001
sprouting angiogenesis	31	33	3.843308	6.07E-05	0.006535	<0.001
regulation of protein tyrosine kinase activity	40	46	3.823879	6.57E-05	0.006956	<0.001

positive regulation of transcription initiation from RNA polymerase II promoter	5	6	3.821247	6.64E-05	0.006917	<0.001
sialyltransferase activity	20	21	3.815319	6.80E-05	0.006973	<0.001
response to interferon-gamma	88	99	3.815148	6.81E-05	0.006869	<0.001
regulation of kidney size	5	5	3.785822	7.66E-05	0.007613	<0.001
steroid binding	61	73	3.750922	8.81E-05	0.008622	<0.001
positive regulation of bone resorption	10	12	3.713883	1.02E-04	0.00984	<0.001
positive regulation of bone remodeling	10	12	3.713883	1.02E-04	0.009695	<0.001
collagen fibril organization	36	36	3.704467	1.06E-04	0.009916	<0.001
glomerulus development	42	44	3.624851	1.45E-04	0.013341	<0.001
adaptive immune response based on somatic recombination of immune receptors built from immunoglobulin superfamily domains	91	228	3.615305	1.50E-04	0.013648	<0.001
calcium-activated potassium channel activity	17	17	3.611891	1.52E-04	0.013637	<0.001
phagocytosis	62	68	3.609953	1.53E-04	0.013551	<0.001
calcium channel activity	88	92	3.589673	1.66E-04	0.014452	<0.001
regulation of calcium ion transport via voltage-gated calcium channel activity	46	47	3.578154	1.73E-04	0.014902	<0.001
regulation of tissue remodeling	37	40	3.553349	1.90E-04	0.016165	<0.001
tissue homeostasis	78	85	3.532565	2.06E-04	0.017264	<0.001
embryonic limb morphogenesis	89	104	3.529207	2.08E-04	0.01726	<0.001
embryonic appendage morphogenesis	89	104	3.529207	2.08E-04	0.017042	<0.001
regulation of homotypic cell-cell adhesion	13	14	3.515712	2.19E-04	0.017707	<0.001
photoreceptor outer segment	32	33	3.507113	2.26E-04	0.018064	<0.001

MHC protein complex	33	46	3.496108	2.36E-04	0.018596	<0.001
regulation of platelet activation	21	22	3.482246	2.49E-04	0.01935	<0.001
acute inflammatory response	54	61	3.478179	2.52E-04	0.019412	<0.001
response to caffeine	12	12	3.47671	2.54E-04	0.019289	<0.001
cyclic-nucleotide phosphodiesterase activity	24	27	3.4661	2.64E-04	0.019833	<0.001
transmembrane receptor protein kinase activity	76	83	3.456761	2.73E-04	0.020297	<0.001
positive regulation of transporter activity	28	30	3.451965	2.78E-04	0.020427	<0.001
sensory perception of temperature stimulus	7	8	3.444503	2.86E-04	0.020763	<0.001
3,5-cyclic-nucleotide phosphodiesterase activity	23	26	3.443682	2.87E-04	0.020595	<0.001
cytosolic calcium ion transport	35	38	3.443463	2.87E-04	0.020385	<0.001
epithelial cell proliferation	45	50	3.433255	2.98E-04	0.020938	<0.001
Rho guanyl-nucleotide exchange factor activity	71	75	3.431285	3.00E-04	0.020864	<0.001
voltage-gated sodium channel complex	13	14	3.407591	3.28E-04	0.02252	<0.001
sodium channel complex	13	14	3.407591	3.28E-04	0.022283	<0.001
positive regulation of cell-substrate adhesion	48	51	3.386791	3.54E-04	0.023793	<0.001
sterol transport	38	41	3.376995	3.66E-04	0.024402	<0.001
cholesterol transport	38	41	3.376995	3.66E-04	0.024153	<0.001
acute-phase response	37	42	3.360967	3.88E-04	0.025341	<0.001
regulation of lipase activity	97	107	3.358244	3.92E-04	0.025336	<0.001
excretion	48	50	3.329992	4.34E-04	0.027774	<0.001
positive regulation of ERK1 and ERK2 cascade	77	83	3.328859	4.36E-04	0.027614	<0.001

regulation of synapse structure and activity	47	49	3.323742	4.44E-04	0.027853	<0.001
protein phosphatase type 2A complex	16	19	3.313376	4.61E-04	0.028628	<0.001
cAMP metabolic process	31	32	3.304465	4.76E-04	0.029272	<0.001
regulation of dendrite development	55	57	3.301381	4.81E-04	0.029317	<0.001
heparan sulfate proteoglycan metabolic process	18	21	3.299158	4.85E-04	0.029274	<0.001
phosphoric diester hydrolase activity	91	98	3.297083	4.88E-04	0.029218	<0.001
calcium ion transport into cytosol	34	37	3.290452	5.00E-04	0.029641	<0.001
positive regulation of ion transmembrane transporter activity	22	23	3.287474	5.05E-04	0.029684	<0.001
positive regulation of protein ubiquitination involved in ubiquitin-dependent protein catabolic process	7	7	3.281417	5.16E-04	0.030056	<0.001
negative regulation of phosphorylation	81	85	3.279335	5.20E-04	0.030008	<0.001
voltage-gated sodium channel activity	14	15	3.26874	5.40E-04	0.030879	<0.001
MHC class II receptor activity	8	11	3.25891	5.59E-04	0.031688	<0.001
integrin-mediated signaling pathway	70	72	3.256359	5.64E-04	0.031696	<0.001
positive regulation of cell growth	78	87	3.247585	5.82E-04	0.032408	<0.001

Table S15. Combined *P* value estimation for ICSNPathway and GSA-SNP by Fisher Stastatics

Candidate pathway	Description	ICSNPathway		GSA-SNP		Combined P value by Fisher's method
		Nominal *P*	FDR	*P*	FDR	
GO:0001750	Photoreceptor outer segment	< 0.001	< 0.001	2.26E-04	< 0.001	3.68E-06
GO:0043498	Cell surface binding	< 0.001	< 0.001	2.88E-05	< 0.001	5.3E-07
GO:0015485	Cholesterol binding	< 0.001	< 0.001	2.27E-08	< 0.001	< 0.001
GO:0051635	Bacterial cell surface binding	< 0.001	< 0.001	9.16E-14	< 0.001	< 0.001

Supplementary Figures

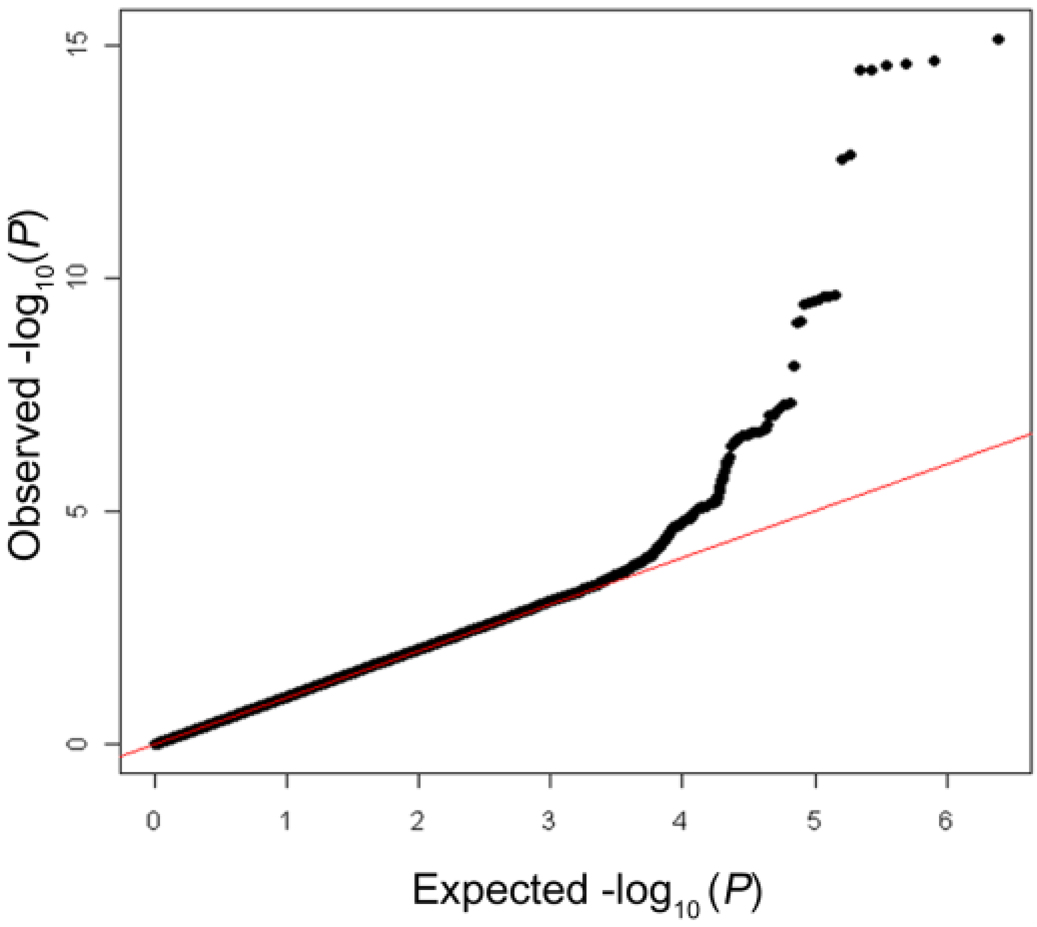

Figure S1. A Quantile–Quantile plot of *P*-values in the GWAS for serum CRP levels (stage 1). The horizontal axis indicates the expected $-\log_{10}$ (*P*-values). The vertical axis indicates the observed $-\log_{10}$ (*P*-values). The red line represents y=x.

A

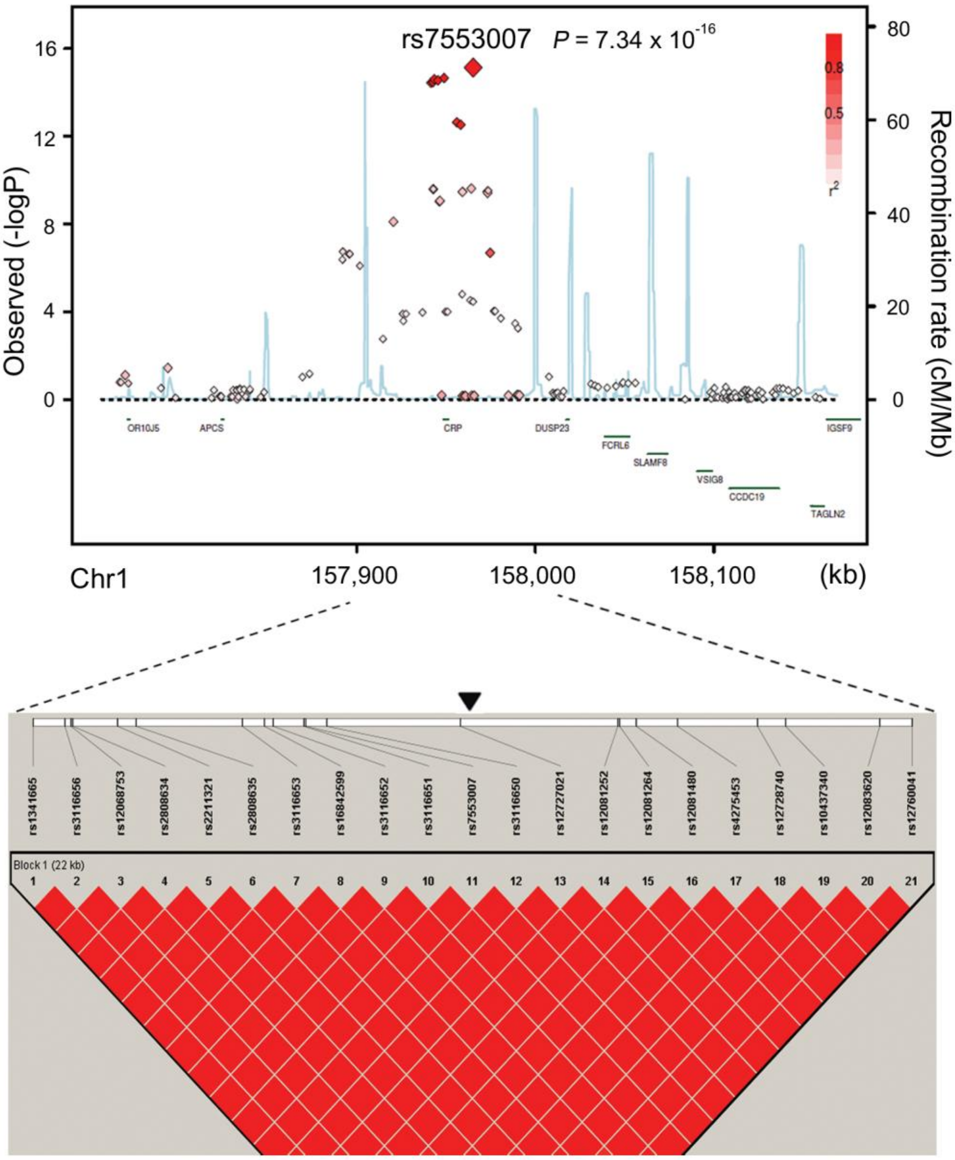
rs7553007 P = 7.34 x 10^{-16}
Observed (-logP)
Recombination rate (cM/Mb)
16
12
8
4
0
80
60
40
20
0
0.8
0.5
r^2
OR10J5
APCS
CRP
DUSP23
FCRL6
SLAMF8
VSIG8
CCDC19
TAGLN2
IGSF9
Chr1
157,900
158,000
158,100
(kb)
rs1341665
rs3116656
rs12068753
rs2808634
rs2211321
rs2808635
rs3116653
rs16842599
rs3116652
rs3116651
rs7553007
rs3116650
rs12727021
rs12081252
rs12081264
rs12081480
rs4275453
rs12728740
rs10437340
rs12083620
rs12760041
Block 1 (22 kb)
1
2
3
4
5
6
7
8
9
10
11
12
13
14
15
16
17
18
19
20
21

B

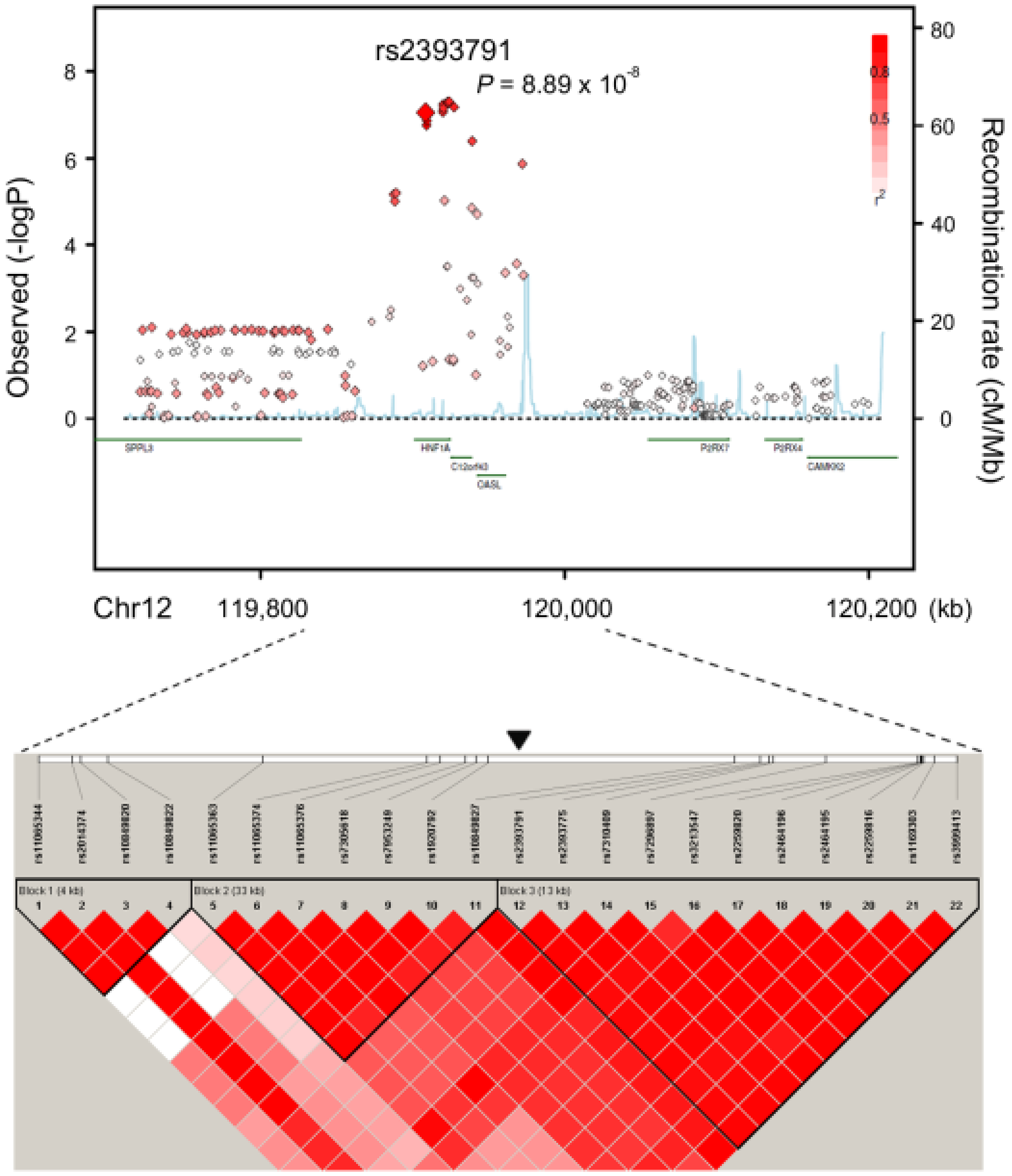

Figure S2. (A) Stage 1 data showing a regional association (upper panel) and linkage disequilibrium (LD; lower panel) plots of the *CRP* locus around rs7553007. Arrow head represents rs7553007. (B) Stage 1 data showing a regional association (upper panel) and linkage disequilibrium (LD; lower panel) plots of the *HNF1A* locus around rs2393791. Arrow head represents rs2393791.

This study

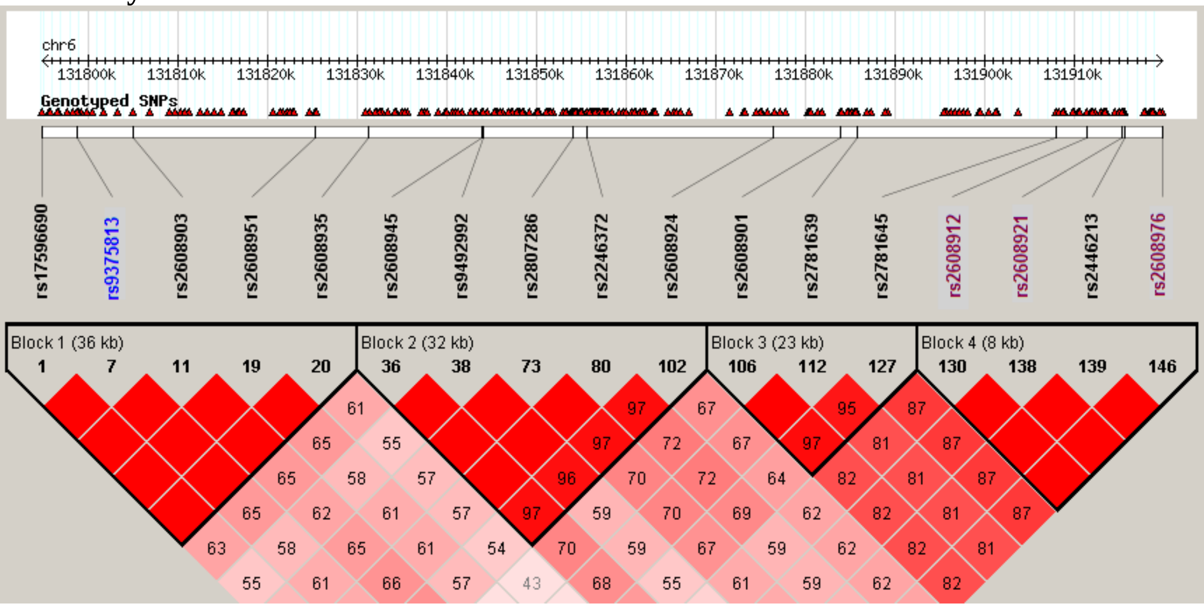

CHB+JPT

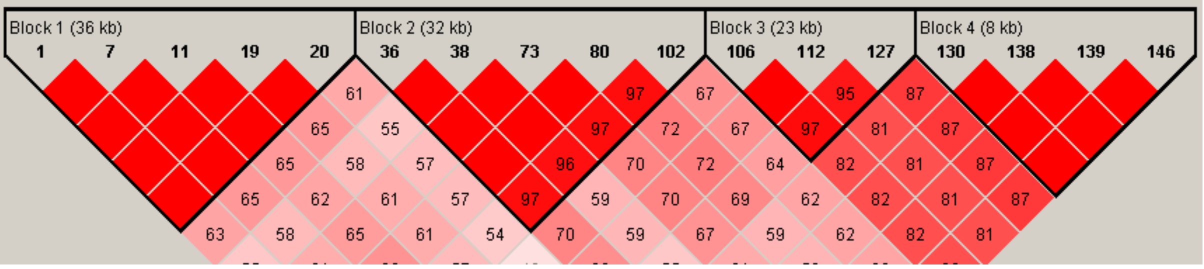

CEU

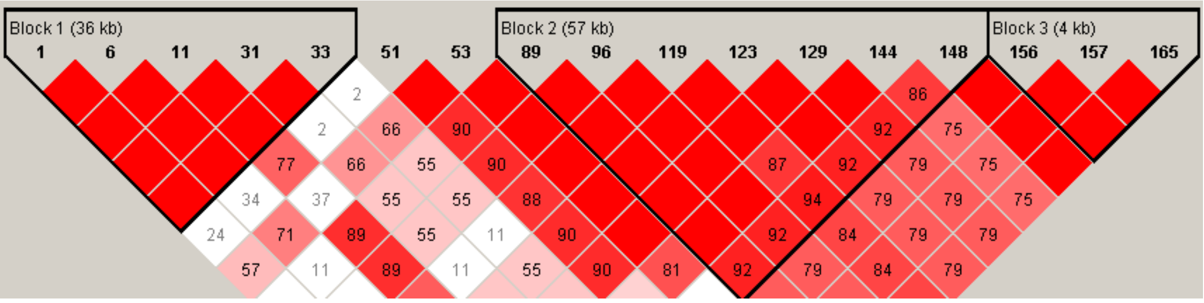

YRI

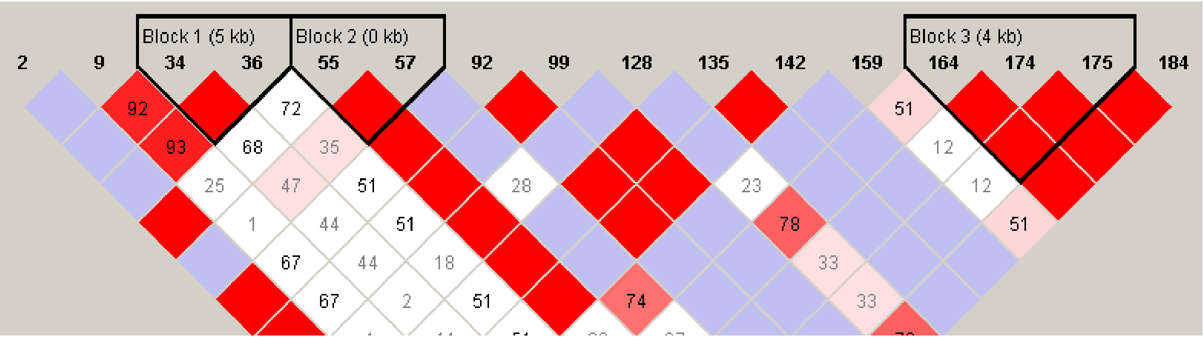

Figure S3. Pairwise linkage disequilibrium (LD) between the selected SNPs in *ARG1* locus around rs9375813. LD plots for Korea population were drawn using the genotype data from the present study, whereas LD plots for Japanese, Chinese, Europeans and Africans were made from genotype data from HapMap Stage 2. Blue ID indicates the most significant SNP. Purple ID indicates the SNPs whose rank got elevated after re-prioritization.

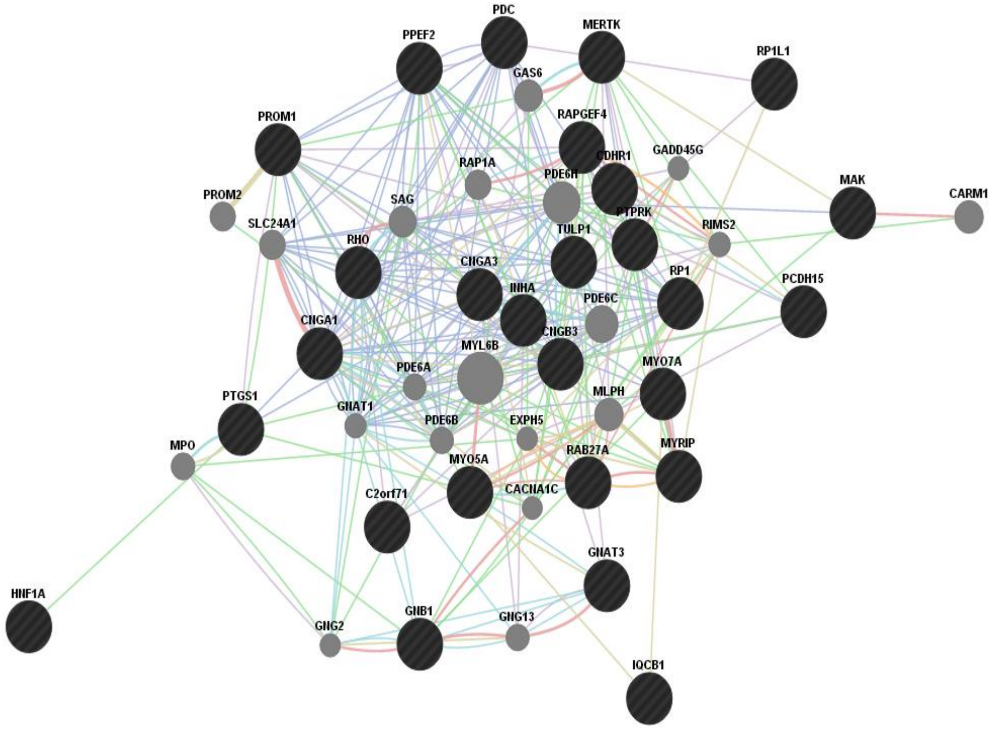

Figure S4. Gene network of photoreceptor outer segment pathway by GeneMANIA analysis. Using the genes identified from pathway analysis, GeneMANIA network analysis was performed. Query genes are depicted as black nodes and discovered genes are depicted as gray nodes. Edges show different interactions among genes; purple indicates for co-expression; light-blue indicates for pathway; dark yellow indicates for shared protein domains; red indicates for physical interactions; dark blue indicates for co-localization; green indicates for genetic interactions. Node sizes are determined according to their weight in the network.

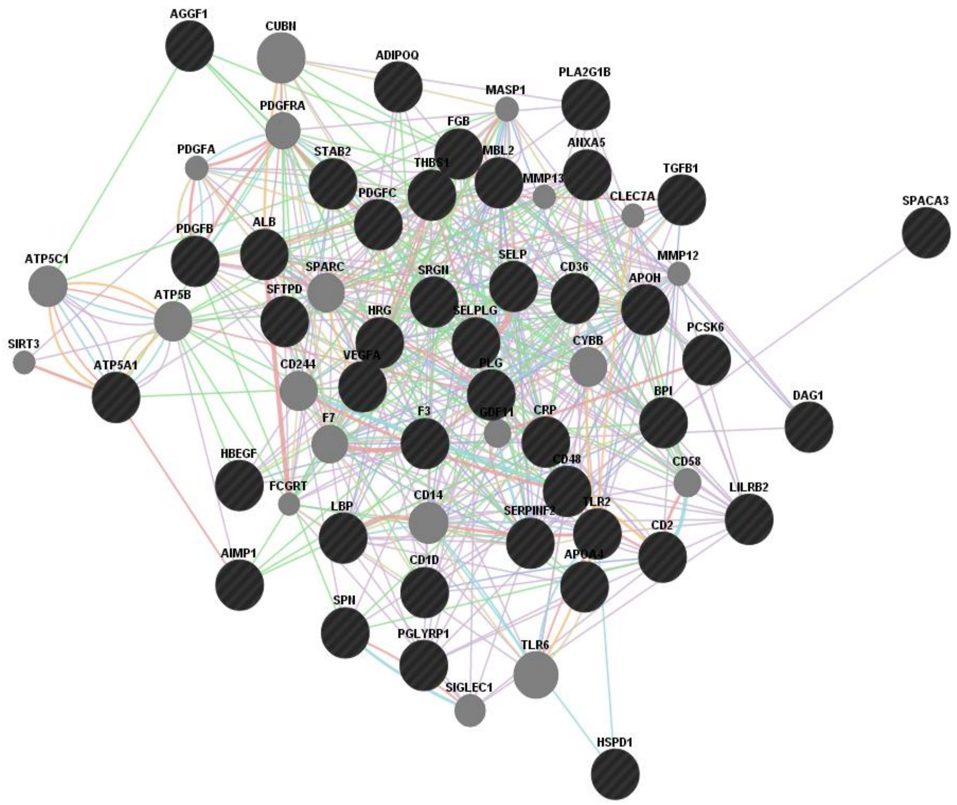

Figure S5. Gene network of cell surface binding pathway by GeneMANIA.

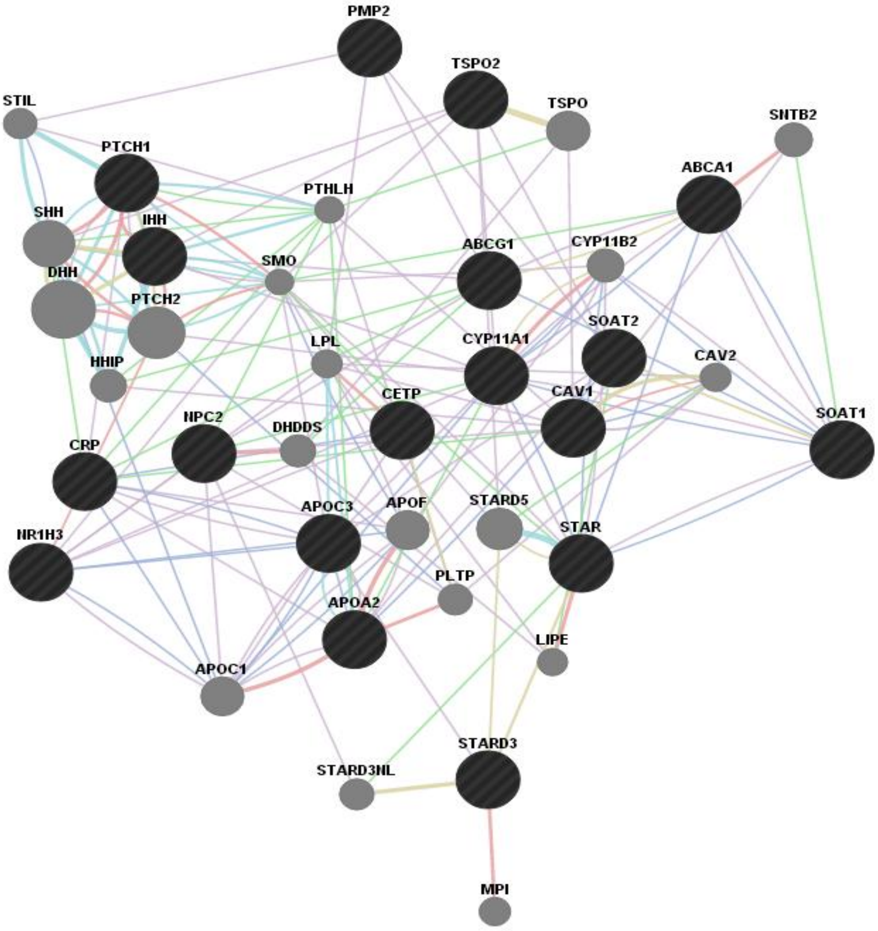

Figure S6. Gene network of cholesterol binding pathway by GeneMANIA.

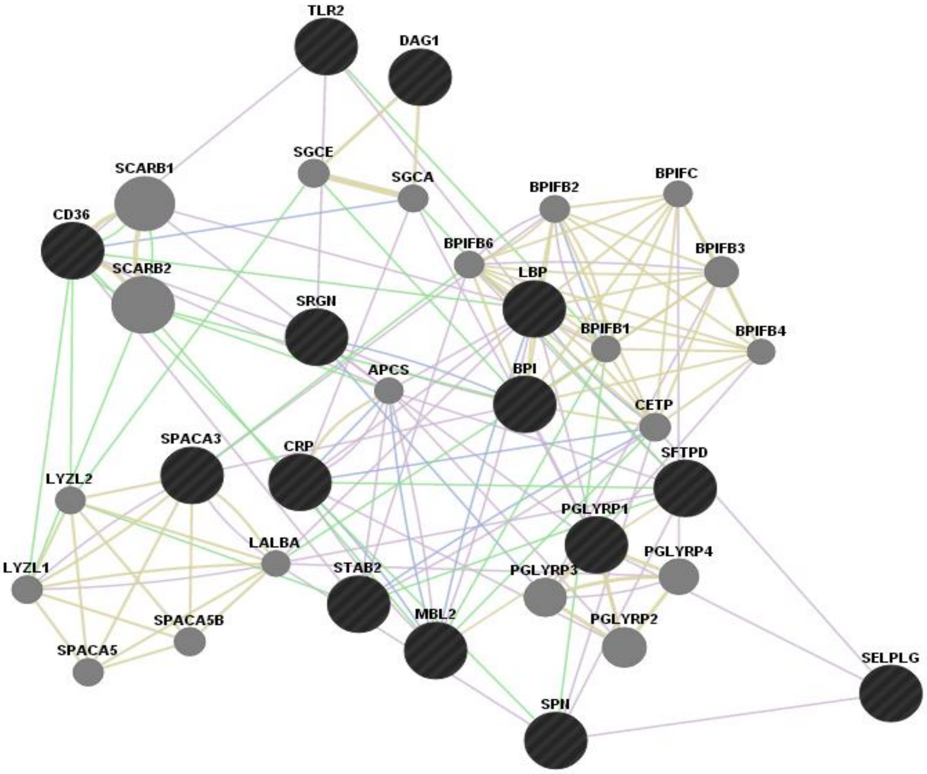

Figure S7. Gene network of bacterial cell surface binding pathway by GeneMANIA.
